# Advanced Dimensionality Reduction for Imaging Mass Spectrometry of Human Eye Tissue through Low-Rank Modeling with Sparse and Dense Residuals

**DOI:** 10.1021/acs.analchem.4c06368

**Published:** 2025-10-13

**Authors:** Roger A. R. Moens, Lukasz G. Migas, David M. G. Anderson, Jeffrey D. Messinger, Olga S. Ovchinnikova, Richard M. Caprioli, Christine A. Curcio, Kevin L. Schey, Jeffrey M. Spraggins, Raf Van de Plas

**Affiliations:** † Delft Center for Systems and Control, 2860 Delft University of Technology, Delft 2628 CD, The Netherlands; ‡ Department of Cell and Developmental Biology, 5718 Vanderbilt University, Nashville, Tennessee 37232, United States; § Mass Spectrometry Research Center, Vanderbilt University, Nashville, Tennessee 37232, United States; ∥ Department of Ophthalmology and Visual Sciences, 9967 University of Alabama at Birmingham, Birmingham, Alabama 35294, United States; ⊥ Center for Nanophase Materials Sciences, 6146 Oak Ridge National Laboratory, Oak Ridge, Tennessee 37831, United States; # Department of Materials Science and Engineering, University of Tennessee, Knoxville, Tennessee 37996, United States; ∇ Department of Biochemistry, Vanderbilt University, Nashville, Tennessee 37232, United States; ○ Department of Chemistry, Vanderbilt University, Nashville, Tennessee 37232, United States; ◆ Department of Medicine, Vanderbilt University, Nashville, Tennessee 37232, United States; ¶ Department of Pharmacology, Vanderbilt University, Nashville, Tennessee 37232, United States; ⋈ Department of Ophthalmology and Visual Sciences, Vanderbilt University, Nashville, Tennessee 37232, United States; ⧓ Department of Pathology, Microbiology and Immunology, Vanderbilt University Medical Center, Nashville, Tennessee 37232, United States

## Abstract

Imaging mass spectrometry (IMS) yields high-dimensional and large data sets commonly exceeding 100,000 pixels, each reporting a mass spectrum of 200,000 intensity values or more. Reducing the dimensionality and size of IMS data is often necessary to enable downstream analysis, and matrix-factorization-based approaches are often used for this purpose. However, the model underlying most of these techniques, decomposing measurements into the sum of a low-rank term (presumed signal) and a small entry-wise residual term (presumed noise), is often not optimal for IMS. For example, while spatially or spectrally sparse signals are common in IMS data, they can heavily distort the low-rank approximation. Therefore, we propose capturing the IMS data structure using low-rank models that, in addition to a dense residual, allow for sparse variation to be captured separately. We implement two such methods, principal component pursuit (PCP) and stable principal component pursuit (SPCP), apply them to IMS data, and compare them to a classical factorization method, principal component analysis (PCA). We investigate their dimensionality and noise reduction performance on MALDI Q-TOF IMS measurements of human cornea and retina tissue since the human eye is a complex organ with lots of small, tightly packed tissue substructures that are spatially sparse. Our results suggest that if parameters are set adequately, PCP and SPCP enable stronger dimensionality reduction and higher compression of IMS data compared to PCA, while concurrently reducing signal overestimation.

## Graphical abstract

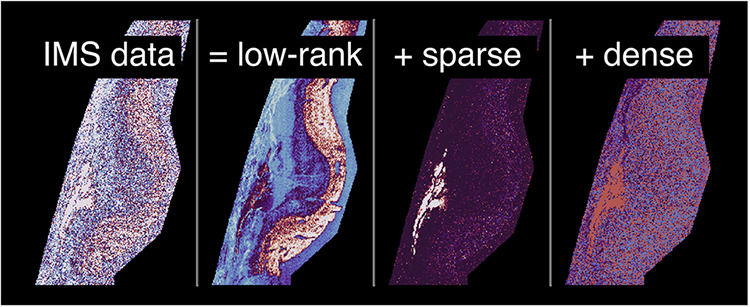

## Introduction

Imaging mass spectrometry (IMS) is an untargeted molecular imaging technique that measures the spatial distributions of a broad set of molecular species concurrently and throughout a sample,^1,2^ with samples including biofilms,^3^ plant material,^4^ mammalian,^5^ and human tissue.^6^ Different instrumental setups are used, with ionization sources ranging from desorption electrospray ionization (DESI)^7,8^ and matrix-assisted laser desorption/ionization (MALDI)^1^ to laser ablation inductively coupled plasma (LAICP)^9^ and secondary ion mass spectrometry (SIMS),^10^ and mass analyzers based on time-of-flight (TOF),^11^ Orbitrap,^12^ and Fourier transform ion cyclotron resonance (FTICR)^13^ principles. While this article focuses on MALDI Q-TOF IMS for tissue samples, the described methods can be applied to other IMS experiment types as well.

MALDI Q-TOF IMS experiments commonly acquire more than 100,000 pixels. Each pixel reports a full mass spectrum, usually entailing more than 200,000 intensity values, resulting in more than 20 billion scalar ion intensity values per experiment. If stored exhaustively, these data set sizes regularly exceed tens to hundreds of gigabytes per experiment. The large size and high-dimensional nature of IMS measurements often necessitate the use of dimensionality reduction techniques to condense the number of dimensions while incurring minimal loss of information. Dimensionality reduction is particularly important for aiding human interpretation of IMS data, denoising, and avoiding issues related to the curse of dimensionality^14^ in downstream computational analysis.

Low-rank approximation is a common approach for IMS dimensionality reduction, utilized, for example, for feature selection, feature extraction, denoising, and visualization of trends that underlie high-dimensional measurements.^15^ Principal component analysis (PCA) and non-negative matrix factorization (NMF) are typical low-rank modeling approaches that have delivered compelling results.^16–18^ However, they often provide suboptimal representations when the IMS measurements contain sparse signals in addition to dense patterns. By sparse signals, we mean noncorrelating signals that only appear in a few mass bins (i.e., spectrally sparse) or in relatively small tissue areas (i.e., spatially sparse). Sparse signals can occur, for example, due to sample preparation or instrument-induced noise perturbations or nonlinear mixing effects in the underlying chemistry, but they can also be genuinely biological in nature due to the sample’s content, structure, and orientation. For example, TOF-SIMS data are sometimes relatively sparse in the spatial domain due to limited ion yields per pixel, while Orbitrap and FTICR data often exhibit high sparsity in the spectral domain due to their high mass resolution and low noise baseline.

We propose capturing such structure inside IMS data more precisely using low-rank models that, in addition to a dense residual, allow for sparse variation to be captured separately. Such models offer advantages over traditional low-rank approaches, including better handling of outliers^19^ and counteracting noise accumulation.^14^ It allows them to deal more effectively with IMS-inherent sparsity, enables closer modeling of the true underlying data structure, and avoids the loss of sparse biological signals in the process. These advantages are essential to approximating IMS measurements with sparse patterns well and to factorizing full-profile mass spectrometry data in general. These models have also been successfully applied in other domains, such as video background subtraction and collaborative filtering, underscoring their broad utility in capturing structured low-rank and sparse variation.

The methods are demonstrated on human eye tissue, specifically the cornea and retina, for dimensionality reduction and noise reduction purposes. These tissues exhibit many small and fine-grained tissue substructures^20^ that report inherently sparse intensity variations and thus are challenging to capture with traditional models (despite reporting genuine biological variation).

This paper (a) explores how low-rank models with sparse residuals, in particular, principal component pursuit (PCP)^19^ and stable principal component pursuit (SPCP),^21^ can be applied to MALDI Q-TOF IMS data, (b) compares their outcomes to traditional approaches such as PCA, and (c) assesses the trade-offs required when modeling. We first introduce the two IMS data sets and the mathematical models behind PCP and SPCP, followed by a discussion of the obtained results and a concluding review of the use cases for this type of model.

## Data Sets and Methods

Two MALDI Q-TOF IMS data sets from the human cornea and human retina tissue sections are used to examine and compare the PCA, PCP, and SPCP dimensionality reduction models (Figures 1 and 2). Figure 1 shows the total ion count spectrum and total ion count image of the cornea data set, and Figure 2 shows the same for the retina data set. A detailed description of these data sets and their preprocessing is provided in the Supporting Information.

**Figure 1:**
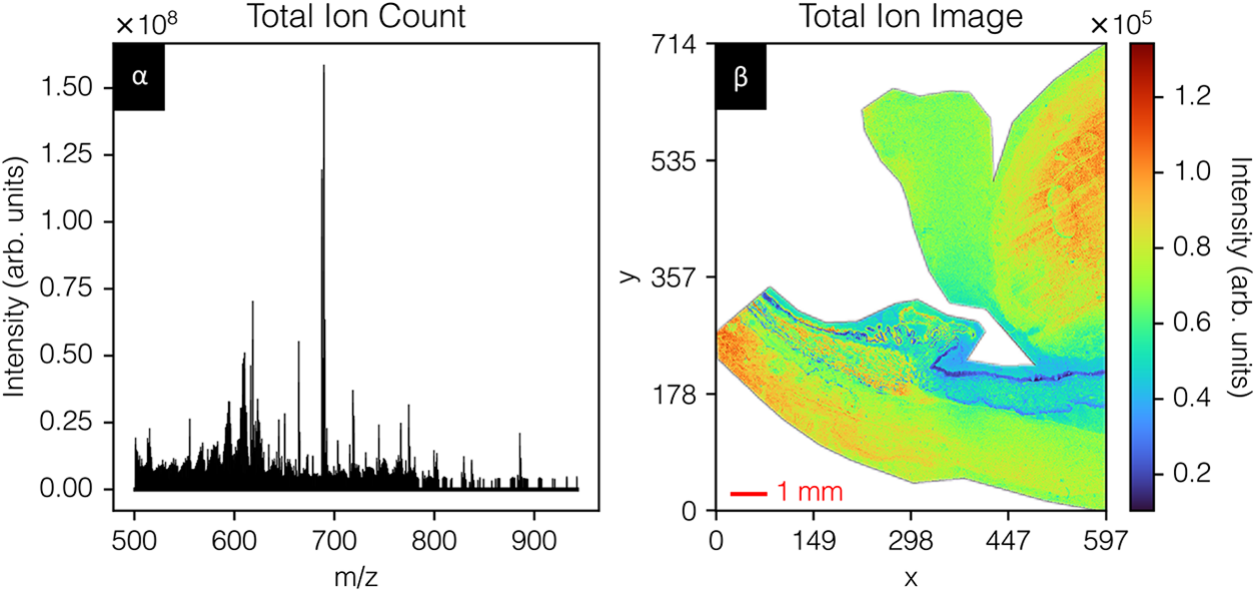
Human cornea data set—total ion count spectrum (panel α) and total ion count image (panel β). The data set entails 235,218 pixels by 2381 peaks. The raw data have been exported, *m*/*z* aligned, calibrated, 5–95% TIC-normalized, and peak-picked. Fine sparse layers, for example, cornea, lens, ciliary processes, and iris, can be observed. An H&E stain is provided in Figure S1.

**Figure 2:**
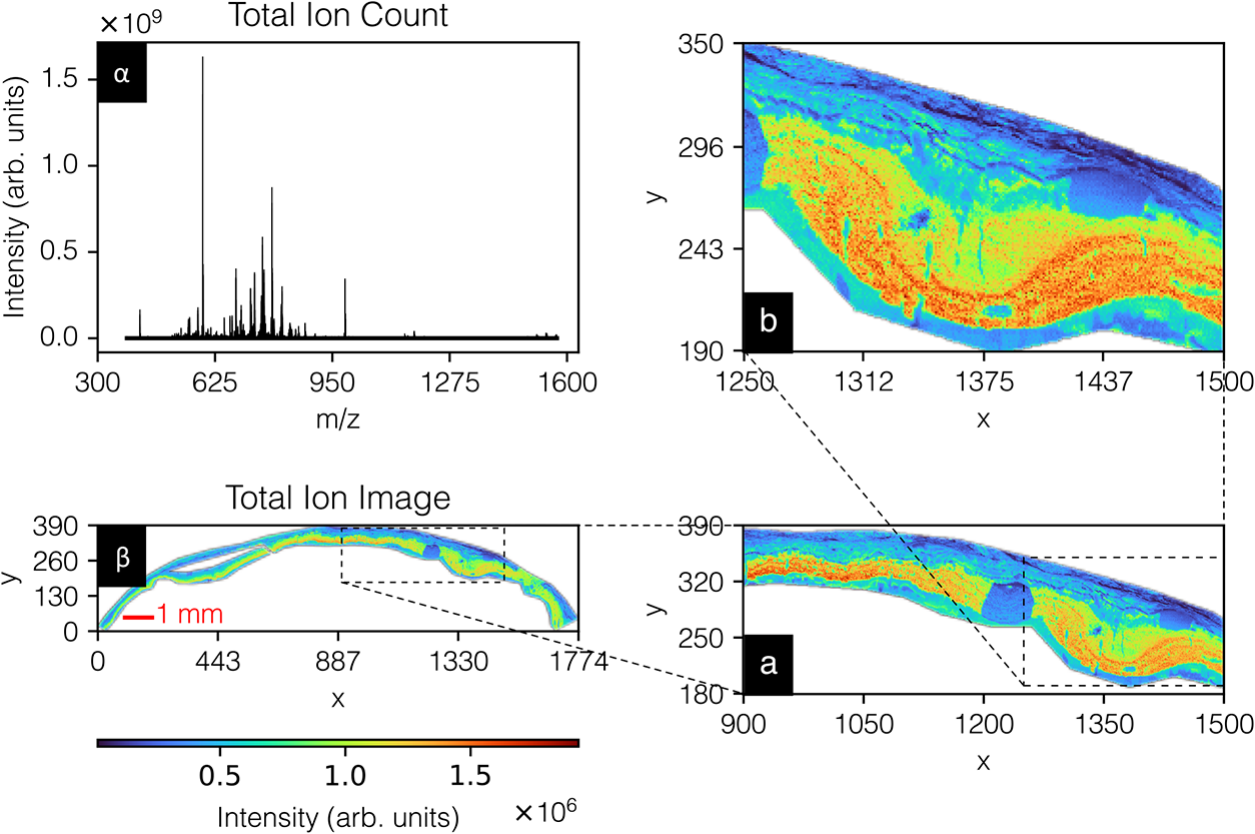
Human retina data set—total ion count spectrum (panel α) and total ion count image (panel β). The data set entails 137,923 pixels by 3212 peaks. The raw data have been exported, *m*/*z* aligned, calibrated, 5–95% TIC-normalized, and peak-picked. Panels (a) and (b) provide zoomed-in views, revealing fine tissue structures, including inner retina, choroid, and sclera. A brightfield image is provided in Figure S2.

### Factorization-Based Dimensionality Reduction Methods

We explore two dimensionality reduction models that extend the low-rank approximation model with an explicit and separate sparse residuals term, and we investigate how these methods fare in terms of capturing the signal from IMS data and avoiding sparse signal loss. The proposed methods are principal component pursuit (PCP)^19^ and stable principal component pursuit (SPCP),^21^ and we compare their results to a more traditional approach, namely, principal component analysis (PCA).^22,23^ The PCA,^22,23^ PCP,^19,24^ and SPCP^21,25^ implementations used in this paper are provided as an open-source Python 3 toolbox[Fn fna].

#### Model Structure

The underlying models of all three methods can be considered members of the family F of extended linear mixture models:^26^

1 $$F:\;A=B+C+D,,\mathrm{with}\;B=YZ^{T}$$

where $A\in R^{m\times n}$ represents a measurement matrix to decompose, and $B\in R^{m\times n}$ , $C\in R^{m\times n}$ , and $D\in R^{m\times n}$ are, respectively, defined as a low-rank term (*B*), a sparse residuals term (*C*), and a dense residuals term (*D*). Furthermore, *B* is a product of factors $Y\in R^{m\times r}$ and $Z\in R^{n\times r}$ , where *r* is the matrix rank of *B*, and it is expected that *r* ≪ min (*m*, *n*). While there exist methods that optimize over *Y* and *Z* explicitly,^27^ we will obtain these factors implicitly by use of a nuclear norm surrogate.

#### Optimizations

The methods can be formulated as optimization problems:

- Principal Component Analysis (PCA),

$f_{\mathrm{PCA}}:R^{m\times n}\times Z_{0}^{+}\to R^{m\times n};\left(A,r\right)\to \left(B\right)$ :

2 $$\begin{array}{ll} \underset{B}{\mathrm{minimize}} & ∥A-B∥\\ \mathrm{subject}\;\mathrm{to} & \mathrm{rank}\left(B\right)\leq r \end{array}$$

- Principal Component Pursuit (PCP),

$f_{\mathrm{PCP}}:R^{m\times n}\times R^{+}\to R^{m\times n}\times R^{m\times n};\left(A,\lambda \right)\to \left(B,C\right)$ :

3 $$\begin{array}{ll} \underset{B,C}{\mathrm{minimize}} & ∥B{∥}_{*}+\lambda ∥C{∥}_{1}\\ \mathrm{subject}\;\mathrm{to} & A=B+C, \end{array}$$

- Stable Principal Component Pursuit (SPCP),

$f_{\mathrm{SPCP}}:R^{m\times n}\times R^{+}$ $\times \;R^{+}\to R^{m\times n}$ $\times \;R^{m\times n};\left(A,\theta ,\delta \right)\to \left(B,C\right)$

4 $$\begin{array}{ll} \underset{B,C}{\mathrm{minimize}} & ∥B{∥}_{*}+\theta ∥C{∥}_{1}\\ \mathrm{subject}\;\mathrm{to} & ∥A-B-C{∥}_{F}\leq \delta \end{array}$$

Our notation uses ∥·∥_1_ as the entry-wise $l_{1}$ -norm, ∥·∥ as the operator norm, ∥·∥_*_ as the nuclear norm, and ∥·∥_*F*_ as the Frobenius norm. The PCA method, *f*_PCA_, has one parameter $r\in Z_{0}^{+}$ and delivers a low-rank matrix *B* = *YZ* ^*T*^ explicitly and a dense residuals term *D* implicitly (as *D* = *A* – *B*). The PCP method, *f*_PCP_, also has one parameter $\lambda \in R^{+}$ and delivers a low-rank matrix *B* = *YZ* ^*T*^ and a sparse residuals matrix *C* explicitly. With PCP, there is no (or only a trivially small) dense residual term *D* since *A* is set to be equal to *B* + *C*. Also note that for PCP, the cardinality (number of nonzero entries, i.e., sparsity) of *C* can be relaxed under certain conditions, such that it can contain dense noise.^28^ The SPCP method has two parameters, namely, $\theta \in R^{+}$ and $\delta \in R^{+}$ . SPCP delivers a low-rank matrix *B* = *YZ* ^*T*^ and a sparse residuals matrix *C* explicitly and a dense residuals term *D* implicitly (as *D* = *A* – *B* – *C*). Further specifics on method conditions are provided in the Supporting Information. While PCA has a computational complexity of O(mnr) , PCP and SPCP involve iterative procedures and *k* repeated SVD computations with estimated complexities of O(kmnr) , making them more computationally demanding but still feasible with GPU acceleration and parallel processing.

#### Parameter Setting

All examined methods have parameters, and a study under inexact recovery conditions^29^ has suggested that, rather than a unique optimal parameter setting, the optima are different for different noise cases. We, therefore, explore method results across a range of potential parameter values. These ranges were chosen on the basis of prior experiments,^29^ are intended to cover a wide array of scenarios, and deliver insight into the impact of parameters on a method’s final result and performance. For PCA, we vary the rank *r* with unit steps from 1 to min­(*m*, *n*). For PCP, a λ-multiplier parameter is varied across 2000 distinct values that are linearly spaced between 0.05 and 3. These values ensure that almost all rank values are represented in the retrieved PCP results, enabling a comparison to PCA and SPCP at a specific rank. The relation to PCP’s λ parameter in eq 3 is $\lambda =\frac{1}{\max\left(m,n\right)}\times \lambda$ -multiplier. For SPCP, a θ-multiplier parameter is linearly varied across 100 values between 0.4 and 2.0. The relation to SPCP’s θ parameter in eq 4 is $\theta =\frac{1}{\max\left(m,n\right)}\times \theta$ -multiplier. Additionally, for SPCP, we vary a σ-multiplier parameter across 100 values, logarithmically spaced between 10^–4^ and 10^–1^. The relation to SPCP’s δ parameter in eq 4 is $\delta =\sqrt{\min\left(m,n\right)+\sqrt{8\min\left(m,n\right)}}∥A{∥}_{F}\times \sigma$ -multiplier.^25^ SPCP’s two parameter ranges result in a grid of 100 × 100 = 10^4^ parameter pairs, explored exhaustively in both IMS case studies.

### Comparison Metrics

When the three methods, *f*_PCA_, *f*_PCP_, and *f*_SPCP_, are applied to the same IMS data set, the differences between their models (eqs 2, 3, and 4) will ensure that each method yields a different decomposition of the same measurements *A*. This means that we will obtain distinct low-rank matrices (*B*_PCA_, *B*_PCP_, and *B*_SPCP_), distinct sparse residuals terms (*C*_PCP_ and *C*_SPCP_, there is no *C*_PCA_), and distinct dense residuals terms (*D*_PCA_, *D*_PCP_, and *D*_SPCP_). Since this paper focuses on dimensionality reduction, we are particularly interested in the low-rank approximation matrix *B* and what is captured by its component vectors in the *Y* and *Z* matrices or, equivalently, by the *U* and *V* components of its singular value decomposition (SVD), that is, *B* = *USV* ^*T*^. We want to assess what the impact is on the low-rank approximation *B* of models that have an explicit sparse residuals term (PCP and SPCP) compared to methods that have no such term (PCA).

To compare different low-rank approximations of the same data set, we formulate two sets of metrics to compare low-rank approximations. The first set of metrics is focused on the content of *B*, trying to capture how much overlap there is between the subspaces captured by PCA, PCP, and SPCP. The second set of metrics is focused on how well the low-rank approximation lines up with physical reality. More precisely, since ion counts are necessarily non-negative data, we can use the negativity of values in *B* as a heuristic for how far a low-rank approximation deviates from physical reality (e.g., which can have an impact on human interpretation). A detailed description of the content-based subspace overlap metrics and the non-negativity metrics can be found in Supporting Information.

## Results and Discussion

We conduct a comparison of PCA, PCP, and SPCP using two MALDI Q-TOF IMS case studies. The first case study is on human eye cornea tissue and is focused on evaluating the dimensionality reduction performance of the different methods, exploring primarily the low-rank approximation term *B* of their decompositions. The second case study utilizes IMS measurements from human eye retina tissue and is focused on evaluating the methods’ noise reduction capabilities with illustrations on specific ion species’ distributions. As such, Case Study 2 primarily examines the sparse residuals term *C* and the dense residuals term *D* of the methods’ decompositions.

### Case Study 1: Dimensionality Reduction in Cornea IMS Data

Reducing the dimensionality of IMS measurements using PCA, PCP, or SPCP is primarily a matter of decomposing the measurements into a sum of *B*, *C*, and *D* terms and subsequently replacing the original matrix *A* by its low-rank approximation *B* for any downstream analysis. In this case study, we compare the low-rank terms provided by the different methods, that is, *B*_PCA_, *B*_PCP_, and *B*_SPCP_. First, we investigate how much difference there is between the low-rank subspaces that the different methods deliver. We do this by calculating the overlap between the retrieved column and row subspaces of the different low-rank terms. Second, we assess the different methods’ tendencies to utilize (nonphysical) negative ion counts to obtain their low-rank approximations. To this end, we compare the *B*_PCA_, *B*_PCP_, and *B*_SPCP_ terms of similar rank in terms of their percentage, sum, and mean of negative entries. The results of this latter analysis can be found in the Supporting Information, Results section.

#### Commonality in PCA-, PCP-, and SPCP-Delivered Subspaces

We depict the results of the particular subspace overlap calculations in the same σ- and θ-multiplier grid used to show the explored parameter space for SPCP (see Supporting Information). Figure 3 shows the commonalities among the PCA-, PCP-, and SPCP-delivered dimensionality-reduced subspaces. Panels (a) and (b) compare the PCP and SPCP approximations of the cornea data set, respectively, reporting the overlap in their captured spatial and spectral patterns. Panels (c) and (d) compare the PCA and SPCP approximations of the cornea data set, respectively, and the overlap in their captured spatial and spectral patterns. The average subspace overlap can be defined as the mean of the singular values of the inner product of the compared subspaces (i.e., *U*_Method 1_^*T*^ *U*_Method 2_ and *V*_Method 1_^*T*^ *V*_Method 2_). Values close to 0 on average signify that different information is captured by the particular orthonormal basis of the low-rank approximations; that is, both spaces tend to be orthogonal with respect to each other. Values close to 1 on average signify that the compared subspaces have overlapping orthonormal bases; that is, information is captured in a similar basis.

**Figure 3:**
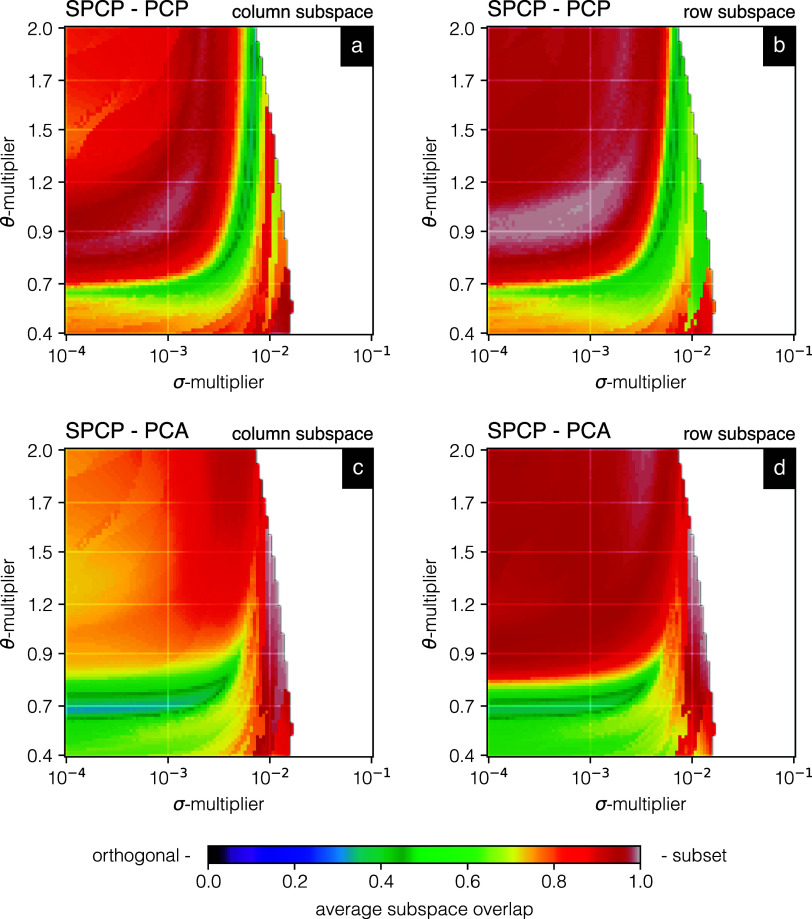
Commonality in PCA-, PCP-, and SPCP-delivered subspaces—average overlap in column and row subspaces between low-rank approximation terms. (a) Overlap between the column subspaces of SPCP (*B*_SPCP_) and PCP (*B*_PCP_), that is, overlap in spatial patterns captured. (b) Overlap between the row subspaces of SPCP (*B*_SPCP_) and PCP (*B*_PCP_), that is, overlap in spectral patterns captured. (c) Overlap between the column subspaces of SPCP (*B*_SPCP_) and PCA (*B*_PCA_), that is, overlap in spatial patterns captured. (d) Overlap between the row subspaces of SPCP (*B*_SPCP_) and PCA (*B*_PCA_), that is, overlap in spectral patterns captured. The average subspace overlap can be defined as the mean of the singular values of the inner product of the compared subspaces (i.e., *U*_Method 1_^*T*^ *U*_Method 2_ and *V*_Method 1_^*T*^ *V*_Method 2_). Values close to 0 on average signify that different information is captured by the particular orthonormal basis of the low-rank approximations; that is, both spaces tend to be orthogonal with respect to each other. Values close to 1 on average signify that the compared subspaces have overlapping orthonormal bases; that is, they are subsets of each other, and similar information is captured on a similar basis.

For SPCP and PCP (Figure 3a,b), gray regions around θ ≈ 0.9 and σ-multiplier ≈ 10^–3^ report almost full overlapping row and column subspaces between SPCP’s and PCP’s solutions, indicating that in that parameter range, SPCP and PCP capture very similar low-rank approximations of the cornea data and describe the captured information in a similar fashion. However, we will note further (see non-negativity metrics) that the SPCP components contain less negative entries than the PCP components. Hence, it is not fully understood why this strong SPCP–PCP overlap appears in this part of the parameter space. Furthermore, in regions of relatively low rank, that is, below 20% (see Figure S3), there seems to be a valley of low overlap (dark green) between the SPCP and PCP approximations. This indicates that a rather large part of the obtained subspaces is orthogonal in this part of the parameter space, suggesting that SPCP and PCP are capturing different aspects of the data there, even though the methods are based on rather similar theory. We note that even though in some regions the overlap is small, the low-rank solutions themselves can still be “close” since we weigh singular values equally, while in the original low-rank approximations, some bases are associated with very large singular values.

For SPCP and PCA (Figure 3c,d), four distinct regions are observed: (i) θ ≈ 0.7, (ii) below θ ≈ 0.7, (iii) above θ ≈ 0.7, and (iv) a region to the right of σ ≈ 10^–2^. Region (i) shows a very low match in column as well as row subspace overlap between SPCP and PCA (dark green), suggesting that in this region of increasing rank SPCP captures a different part of the spatial and spectral space of the IMS data than PCA does. Region (ii) exhibits a poor match between the PCA- and SPCP-delivered column and row subspaces, indicating that there are quite large differences between the dimensionality-reduced subspaces delivered by these methods at low rank. Region (iii) shows a good match in the row subspace but a poor match in the column subspace, indicating that while similar spectral trends are discerned by PCA and SPCP, their spatial component images are quite different. This could indicate that spatially sparse (and highly intensive) features in the tissue, which we know to be present in this data set, are captured by SPCP in its residuals terms, while PCA has no choice but to capture such variation in its low-rank term, causing the component images to deviate substantially. Finally, the gray coloring in region (iv) suggests an almost complete match between SPCP and PCA, which is probably due to the very low rank in that area not allowing much differentiation between the methods.

Overall, we are able to compare the content of the dimensionality-reduced approximations delivered by PCA, PCP, and SPCP. We can distinguish different subareas within the parameter space where the approximating subspaces from the different methods are overlapping and others where the subspaces are very different from each other. Between SPCP and PCP, we can observe a large overlap for most parameter settings, suggesting information is captured on a similar basis, and thus, similar underlying trends are found by these methods. Between SPCP and PCA, we observe that while the row subspaces provided by these methods are quite similar, the overall column subspace overlap seems to be lower. This indicates that, while the underlying spectral patterns captured are similar, the corresponding spatial images indicating where these spectral patterns are active in the tissue are quite different between PCA and SPCP. One possible explanation could be that SPCP, in providing a sparse residuals term *C*_SPCP_, offers a place for spatially sparse features in the data to go rather than into its low-rank approximation term *B*_SPCP_. Since PCA has no such sparse residuals term, spatially sparse features in the tissue can only be captured in the low-rank approximation *B*_PCA_, driving up the necessary rank to approximate a measurement set and modifying the spatial signatures of the principal components if the rank is set. This suggests that spatially sparse patterns in IMS data could substantially skew (at least the component images of) the low-rank approximation delivered by PCA, an issue SPCP does not suffer from. It should also be noted that if a spatially sparse tissue pattern ends up in the sparse residuals term in SPCP, that does not mean it is considered noise and is thrown away. It just means that SPCP’s low-rank approximation has the option to disregard spatially sparse patterns (which could be considered spatial outliers from a global pattern perspective) and thus is able to model the global data patterns more tightly and robustly than PCA.

### Case Study 2: Noise Reduction in Retina IMS Data

The second case study moves focus from the methods’ dimensionality reduction to their noise reduction capabilities. This means that in the context of the family F of extended linear mixture models (eq 1), our attention shifts from the low-rank approximation term *B* (in Case Study 1) to the sparse residuals term *C* and dense residuals term *D* for Case Study 2. We explore the residual terms delivered by PCA, PCP, and SPCP for five particular parameter settings (see Case Study 2 description in Supporting Information).

#### Ion Intensity Distribution among Terms

The global entry-wise intensity histograms (representing values across all *m*/*z* bins) for the *B*, *C*, and *D* terms delivered by PCA, PCP, and SPCP are shown in Figure S9. These allow us to observe the impact of the θ- and σ-multipliers on the spread of the measurements’ energy in *A* among the different decomposition terms. A detailed set of observations for each of the five cases is provided in the Supporting Information.

Across all cases, the residuals term *D* of PCA (PCA has no *C* term) shows quite dense intensity histograms (i.e., lots of nonzero values spread across a broad range of intensity levels; in gray). The residuals term *C* of PCP (PCP has no *D* term) also shows relatively wide and dense intensity histograms (i.e., lots of nonzero values populating a broad range of intensity levels, in red). However, for SPCP, which has both *C* and *D* terms, we see the communication between the sparse and dense residual terms in action. In all five cases, whenever the dense residuals term *D*_SPCP_ is allowed to siphon off dense variation that is not very structured (so not easily captured by *B*) and not very sparse (so not easily captured by *C*), it allows SPCP’s sparse residuals term *C*_SPCP_ to capture a more sparse intensity distribution (see blue traces in *C* column of Figure S9) than PCP’s sparse residuals term *C*_PCP_ (see red traces in *C* column of Figure S9). This is readily visible in Case 3 where the blue *C*_SPCP_ histogram contains less energy than the red *C*_PCP_ histogram and reports a smaller number of high-intensity peaks, indicating sparse intensity distributions. Presumably, the “escape valve” offered by SPCP’s dense residuals term also allows the low-rank approximation *B* to capture a tighter low-rank model of the data, but this is a hypothesis and is hard to assess from the intensity histograms in this figure.

This assessment of how ion intensity variation is distributed among decomposition terms shows that unlike PCA and PCP, at least for retina data, SPCP has the ability to (a) obtain a low-rank representation of an IMS data set, which is useful for dimensionality reduction, compression, and interpretation, (b) capture spatially and spectrally sparse features in the data, which are not necessarily noise but would require a lot of extra dimensions if they were forced to be represented by the low-rank approximation, and (c) separate out a layer of low-intensity dense variation, which can usually be considered noise and be thrown out. SPCP performs this decomposition of measurements in one optimization run and delivers the different signal components concurrently to the user, going beyond what PCA and PCP can deliver for the same data set.

#### Ion Species-Specific Effects

After examining what PCA, PCP, and SPCP do with the content of an IMS measurement set on a data set-wide scale, we now investigate how SPCP’s parameter setting influences individual ion species, mass bins, or ion images. Figure S10 shows the distribution of energy among the *C* and *D* terms for six distinct *m*/*z* bins: *m*/*z* 601.53 (ion image with sparse spatial structures); *m*/*z* 1007.01 (low-intensity ion image); *m*/*z* 790.52 (low to average-intensity ion image); *m*/*z* 666.43 (average intensity ion image); *m*/*z* 554.57 (high-intensity ion image without strong outliers); *m*/*z* 591.01 (high-intensity ion image with strong outliers). Each panel shows the (histogram-ed) content of the residuals terms of PCA, PCP, and SPCP for a specific parameter setting/decomposition (Cases 1 through 5 specified in the Supporting Information) and for a specific *m*/*z* bin. The measured variation that ends up in the residuals terms *C* and *D* is the difference between the raw measurement *A* and its low-rank low-dimensional representation *B*. If one considers the low-dimensional representation as the ‘important’ part of the data set, the content of *C* and *D* could potentially be labeled as the ‘nonimportant’ part of the measurement and thus could be removed to effectively ‘denoise’ the measurement. The dense residuals term *D* is not optimized toward a particular type of content, simply capturing whatever is not already represented by the other terms in the decomposition, so in almost all use cases, the removal of *D* is a useful denoising technique. The sparse residuals term *C* is optimized toward capturing sparse features in the measurements, which are not necessarily noise, so whether or not to remove *C* to denoise is an application-specific consideration conditioned on whether we think that the sparse signal should be retained for downstream analysis or not. A more detailed treatment of the histogram traces in Figure S10 is provided in the Supporting Information.

We observe that under certain parameter settings, the residuals of PCA, PCP, and SPCP for a specific *m*/*z* bin can be very similar, for example, *m*/*z* 1007.01 and *m*/*z* 790.52 for Cases 2 and 3, suggesting a similar denoising capability for those ion species. However, this behavior seems primarily correlated to low- or low-to-average-intensity ion species. When examining high-intensity ion species (*m*/*z* 666.43 and 554.57) or ion distributions that exhibit sparse signals (*m*/*z* 591.01), much larger differences between the methods can be discerned. We also see that in most *m*/*z* bins, the residuals of PCP and SPCP contain inherently less negative entries, which correspond to our findings in Case Study 1. Finally, Figure S10 shows that PCA’s residual intensity distribution is mostly symmetric around the origin, where the PCP and SPCP residuals exhibit mostly asymmetric intensity distributions. Different parameter settings lead to different residual distributions local to specific ion species, and some of these effects are tied to the nature of the ion species’ intensity level (low versus high-intensity ions) and spatial distribution (e.g., sparse features or not). This suggests that the parameters for PCA, PCP, and SPCP could and should be optimized differently in function of whether one wants to amplify or attenuate such ion species-specific effects. Overall, residuals of PCP and SPCP tend to contain less negative intensity values, suggesting less overestimation of the mass spectral signals in these methods’ low-rank approximations.

#### Effects on Images

While the histogram view on the distribution of measured ion counts among the different components gives a broad view into what is happening when a PCA-, PCP-, or SPCP-based dimensionality reduction is applied to IMS data, the final assessment comes down to what these methods mean in terms of the (denoised) images that they provide. Specifically, we explore how each of these methods decomposes the same measured ion image into a low-rank approximated image, a sparse variation image, and a dense residual image. Figure S11 shows for the six ion species examined in the previous section what ends up in their sparse (i.e., *C*) and dense (i.e., *D*) residual images for five different parameter settings, and it allows us to make some general observations. The original ion images can be found in Figure S8.

For PCA, it is expected that the *D* term captures small entry-wise noise (see Data Sets and Methods). However, we observe large negative entries as well, for example, dark blue pixels in Cases 2 and 3. The fact that PCA’s *D* term is trying to negatively compensate against its *B* term to arrive at the measured ion intensity could potentially mean that the estimated signal in PCA’s low-rank approximation *B* term is overestimated (i.e., false signal creation in the low-rank term). This is generally undesirable since it could suggest tissue structure in the (denoised) *B* image that is not really present. We also observe genuinely biological sparse tissue features in PCA’s sole residuals term, for example, small dark red colored areas for *m*/*z* 601.53 and 591.01. This suggests that if PCA’s *D* term is simply labeled noise and thrown away without inspection, genuine biological information could be lost in the process.

For PCP, it is expected that its *C* term captures sparse residuals, which could be noise or a sparse biological signal. However, since the PCP model (eq 3) has only two terms to represent measured ion intensity, its *C* term also needs to capture whatever is not already represented by PCP’s low-rank approximation term *B*, which means it has to occasionally capture dense noise patterns as well. In Figure S11, we observe less negative entries (blue) in PCP’s *C* than in PCA’s *D*, suggesting less opportunity for overestimation in the low-rank approximation of PCP compared to that provided by PCA at the same rank. We observe more high-intensity positive (red) sparse features in PCP’s *C* than in PCA’s *D*, yet we do not see many truly sparse images (mostly or almost fully yellow, with the occasional sparse red feature) in PCP’s *C*. This might be problematic when sparse features are under investigation, and PCP’s model is simply assumed to deliver a direct notion of those features in its *C*, since this is shown to not necessarily pan out.

The problem of PCP’s *C* term not necessarily capturing sparse variation is largely solved by SPCP’s model (eq 4). SPCP can dedicate its *C* term to capturing sparse noise and sparse biological signal by providing an extra *D* term to capture small entry-wise noise. This is reflected in Figure S11’s bottom two panels. We see SPCP’s *C* images capture primarily sparse red features, for example, in Cases 2, 3, and 4, among an image that consists largely of yellow pixels close to zero. We also observe that SPCP’s *D* term captures dense noise, for example, in Cases 1, 4, and 5, while for other cases, it will contain negative entries, for example, for *m*/*z* 666.43, 554.57, and 591.01. The discrepancy between PCP’s *C* and SPCP’s *C* seems to suggest that SPCP is better suited for capturing genuine sparse variation in the data. We therefore conclude that SPCP, in contrast to PCA and PCP, is able to factor out sparse signals more straightforwardly, but that parameter tuning is crucial for optimally exploiting that sparsity and for minimizing possible signal overestimation.

While we have discussed the content of the different decomposition terms in isolation, it is valuable to bring these different aspects of a measured ion image together for a specific ion species to see how the different dimensionality reduction methods, PCA, PCP, and SPCP, decompose the same ion image. Figure 4 shows, for ion species *m*/*z* 601.53, the measured ion image (*A*), the low-rank approximation image (*B*), the sparse residuals image (*C*), and the dense residuals image (*D*). This ion species is an example where the ion distribution contains a genuinely biological sparse tissue feature, and we see where this pattern ends up for filtering or retention. Figure 5 shows the same results for ion species *m*/*z* 1007.01, whose ion distribution does not contain sparse tissue features. The strong similarity in low-rank approximations between PCA, PCP, and SPCP when no sparse variation is present in the data (e.g., *m*/*z* 1007.01) and PCP and SPCP’s clear separation of sparse variation when sparse patterns are present in the data (e.g., *m*/*z* 601.53) provide some guidance for why PCP and SPCP-based dimensionality reduction should be considered for IMS data, a data type where sparse variation is often prominently present.

**Figure 4:**
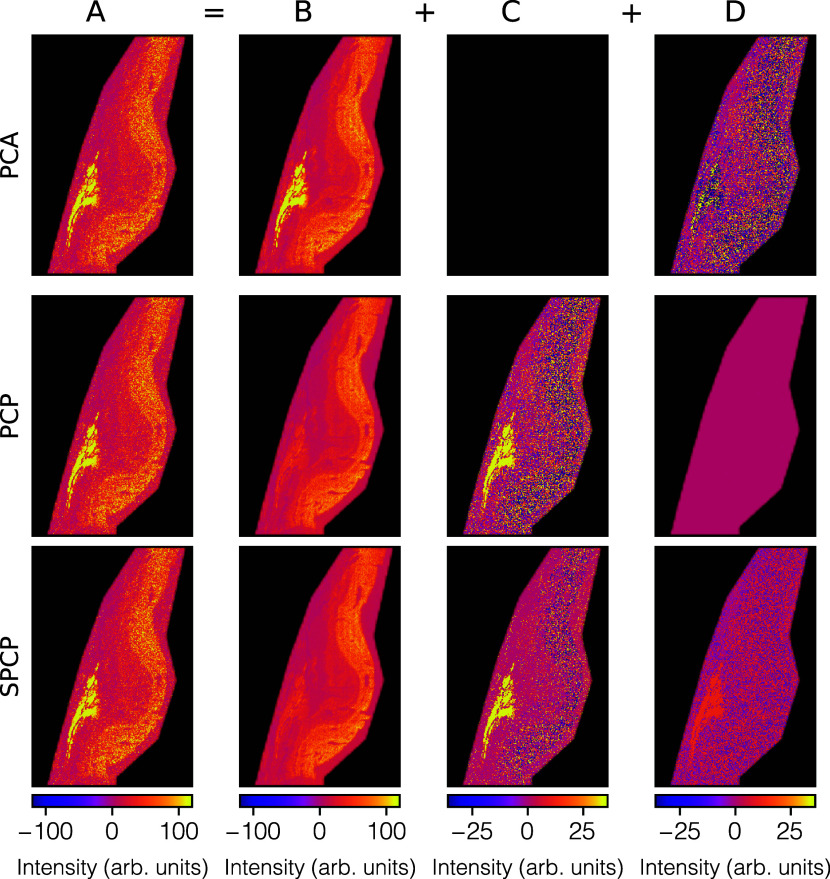
PCA, PCP, and SPCP decompositions of *m*/*z* 601.53’s measured retina ion image (A) into the sum of a low-rank approximation image (B), a sparse residuals image (C), and a dense residuals image (D). We observe an example of a sparse tissue structure (in bright yellow) in ion image A. In this figure, A, B, C, and D show one column from, respectively, matrices A–D, refolded into an image. Note, however, that the methods have been applied to the whole set of ion images in one go (see Supporting Information, Data Preprocessing). In PCA, this feature needs to be captured in the low-rank approximation image B, either costing additional dimensions or skewing the axes of the lower-dimensional space to accommodate this high-intensity feature. In PCP and SPCP, the sparse tissue feature ends up primarily in the sparse residual image C, where a call can be made whether one wants to remove or retain that sparse content. If the latter, SPCP’s C image tends to be cleaner since it can funnel off dense noise to its dense residuals image D, allowing for dense noise removal even when sparse patterns are being retained. While at first it might seem that PCP and SPCP’s low-rank approximation image does not leave much trace of the sparse tissue feature, it is actually still relatively well captured by those images if one takes the different color maps into account. Even if C is removed and only B is retained, the tissue feature would still be present in the lower-dimensional representation of the data, albeit at less high ion intensities.

**Figure 5:**
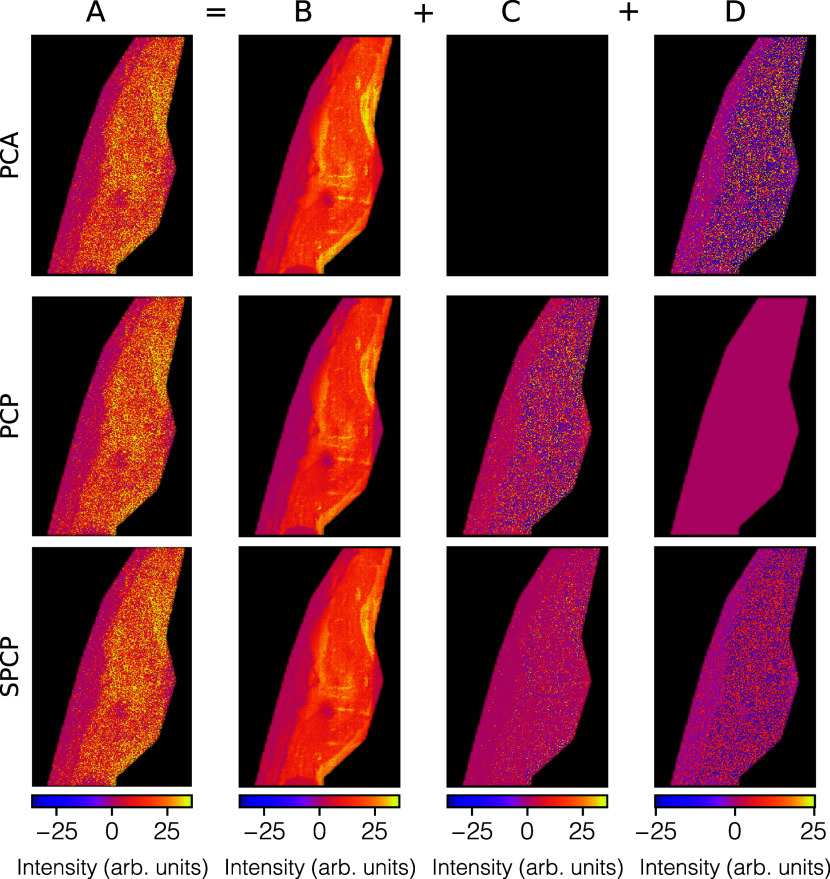
PCA, PCP, and SPCP decompositions of *m*/*z* 1007.01’s measured retina ion image (A) into the sum of a low-rank approximation image (B), a sparse residuals image (C), and a dense residuals image (D). In this figure, A, B, C, and D show one column from, respectively, matrices A through D, refolded into an image. Note, however, that the methods have been applied to the whole set of ion images in one go (see Supporting Information, Data Preprocessing). This ion species is an example of an ion distribution without many sparse tissue features and demonstrates the strong similarity between PCA, PCP, and SPCP results when sparse variation is absent.

In general, the low-rank approximation image *B* tends to capture the global tissue structure underlying the sometimes noisy ion image *A*. Using image *B* for downstream analysis rather than image *A* would effectively amount to denoising the ion image and potentially revealing tissue structure that might go unrecognized if it remained buried among noise. The dense residuals image *D* tends to capture unstructured variation across the whole data set, is generally labeled as noise, and thus tends to be removed from further analysis. The sparse residuals image *C* tends to capture the sparse variation in the measured ion image. Such sparse variation could be noise, but if spatially or spectrally sparse signals are present in the IMS data (e.g., due to fine low-pixel-area tissue structures), that sparse variation could be genuinely biological in nature. The advantage of capturing such sparse variation as a separate image is that the user has the option to inspect the image and assess whether the sparse variation seems to delineate biological patterns. If the image is deemed biological in nature, the user has the option to retain the sparse image and add it back to the low-rank image for downstream analysis. If it is deemed noise or it is not informative, the sparse image can be removed from further analysis. Regardless of whether the sparse image is retained, the ability to separate it in the PCP and SPCP cases and avoid sparse variation from needing to be captured by the low-rank approximation allows for tighter modeling of the underlying trends, a reduction in the number of dimensions that need to be retained (so better compression) and generally more physically interpretable axes for the lower-dimensional subspace.

## Conclusions

We introduced and compared PCP and SPCP as alternatives to the more traditional PCA dimensionality reduction (and noise reduction) method for IMS measurements. Using a cornea data set, we investigated the commonality among PCA-, PCP-, and SPCP-delivered subspaces and their low-rank approximations. We also assessed how far an added sparse residuals term *C*, as in the PCP and SPCP decompositions, impacts the non-negativity of these methods’ low-rank approximations, and thus, the physical feasibility and interpretability of the components and underlying trends retrieved from the data. In a retina case study, we explored the global as well as ion species-specific distribution of measured ion intensity among the different decomposition terms and the influence of parameter settings on these distributions.

Our initial observations suggest that SPCP allows for a higher compression by separating out sparse residuals into a separate term, thus requiring fewer components to describe the remaining data. SPCP also seems to deliver more physically interpretable components for non-negative data than PCP and PCA. This was demonstrated on the cornea data set, especially for data approximations of lower rank. The overlap in row and column subspaces of the low-rank approximations between all methods suggests that information is captured on a similar spectral basis. However, we could observe substantial differences in subspace overlap along the spatial domain, which might be an indicator of genuinely sparse biological features being more common on the spatial rather than spectral side for our cornea tissue data. From our second case study on retina data, we can conclude that the parameter setting is highly influencing the recovered terms. For example, it shows that setting the σ-multiplier in combination with the θ-multiplier for SPCP can have a large effect on the cardinality of the sparse term. The latter is useful if sparse features are of interest. We can also conclude that SPCP and PCP, in contrast to PCA, have a tendency to approximate the signal “from below,” that is, by a smaller value, minimizing possible signal overestimation.

This work demonstrates the beneficial capabilities of more advanced factorization methods in capturing approximations of (I)­MS data. This can be a first step toward enabling full mass-profile factorization and analysis at scale. At the same time, we have shown that these advanced methods require more and better fine-tuning of parameters to find the appropriate results. The latter is of increasing difficulty due to growing data set sizes. In conclusion, our research has shown that exploiting sparsity is useful yet underappreciated in IMS data analysis and that it might be a key consideration for future dimensionality reduction solutions for IMS data and other molecular imaging modalities.

## Supplementary Material



## Footnotes

a
https://github.com/vandeplaslab/hannibalspecter

## Notes

Additional details and figures related to the data sets, methods, case studies, comparison metrics, and case study results (PDF)
