# Supplementary material for: Advanced Dimensionality Reduction for Imaging Mass Spectrometry of Human Eye Tissue through Low-Rank Modeling with Sparse and Dense Residuals
Source: Anal Chem. 2025 Oct 13;97(42):23040–9. doi: 10.1021/acs.analchem.4c06368 (PMC12573230; doi:10.1021/acs.analchem.4c06368)
Supplement: Supplementary file 1 [file ac4c06368_si_001.pdf]

Supporting Information Available

Supplementary Information

for

Advanced Dimensionality Reduction for Imaging Mass Spectrometry of Human Eye Tissue Through Low-Rank Modeling with Sparse and Dense Residuals

Roger A.R. Moens[1], Lukasz G. Migas[1], David M.G. Anderson[2,3], Jeffrey D. Messinger[4], Olga S. Ovchinnikova[5,6], Richard M. Caprioli[3,7,8,9,10], Christine A. Curcio[4], Kevin L. Schey[3,7,8,11], Jeffrey M. Spraggins[2,3,7,8,12], and Raf Van de Plas[1,3,7]

1. Delft Center for Systems and Control, Delft University of Technology, Delft 2628 CD, Netherlands.
2. Department of Cell and Developmental Biology, Vanderbilt University, Nashville, TN 37232, USA.
3. Mass Spectrometry Research Center, Vanderbilt University, Nashville, TN 37232, USA.
4. Department of Ophthalmology and Visual Sciences, University of Alabama at Birmingham, Birmingham, AL 35294, USA.
5. previously Center for Nanophase Materials Sciences, Oak Ridge National Laboratory, Oak Ridge, TN 37831, USA.
6. Department of Materials Science and Engineering, University of Tennessee, Knoxville, TN 37996, USA.
7. Department of Biochemistry, Vanderbilt University, Nashville, TN 37232, USA.
8. Department of Chemistry, Vanderbilt University, Nashville, TN 37232, USA.
9. Department of Medicine, Vanderbilt University, Nashville, TN 37232, USA.
10. Department of Pharmacology, Vanderbilt University, Nashville, TN 37232, USA.
11. Department of Ophthalmology and Visual Sciences, Vanderbilt University, Nashville, TN 37232, USA.
12. Department of Pathology, Microbiology and Immunology, Vanderbilt University Medical Center, Nashville, TN 37232, USA.

E-mail: raf.vandeplas@tudelft.nl

Contents

- **Supplementary Information to the Datasets** – page S3
  - **Sample Preparation** – page S3
  - **MALDI Q-ToF IMS Datasets** – page S4
  - **Data Preprocessing** – page S5
  - **Micrographs** – page S6
- **Supplementary Information to the Methods** – page S7
  - **Method Conditions** – page S7
- **Supplementary Information to the Case Studies** – page S7
  - **Case Study 1: Focus on Dimensionality Reduction** – page S7
  - **Case Study 2: Focus on Noise Reduction** – page S11
- **Supplementary Information to the Comparison Metrics** – page S12
  - **Content-based Metrics: Metrics Reporting overlap in Recovered Column and Row Subspaces** – page S12
  - **Non-negativity Metrics** – page S14
- **Supplementary Information to the Results of Case Study 1** – page S15
  - **Negative Entries in the Low-Rank Approximation** – page S15
  - **Percentage of Negative Entries** – page S16
  - **Sum of Negative Entries** – page S18
  - **Mean of Negative Entries** – page S18
- **Supplementary Information to the Results of Case Study 2** – page S20
  - **Ion Intensity Distribution Among Terms** – page S22
  - **Ion Species-Specific Effects** – page S25

Supplementary Information to the Datasets

This section describes the sample preparation protocols for the retina and cornea tissues, acquisition details of the instrumentation used for MALDI Q-ToF IMS measurement, dataset characteristics, and preprocessing steps applied before analysis.

Sample Preparation

The following is based on a description by the same co-authors in Anderson et al.[1]. The retina tissue sections were prepared as described by Anderson et al.[2]. Whole eyes were obtained from deceased human donors by Advancing Sight Network (Birmingham, AL) as part of ongoing studies on age-related macular degeneration (AMD) that are approved by institutional review at University of Alabama at Birmingham (protocol # N170213002), where tissues were collected. Whole globes were opened anteriorly and immersed in 4% phosphate-buffer paraformaldehyde (PFA) overnight. Globes were then placed in 1% PFA at 4°C for up to 48 h prior to sectioning. Dissected tissue containing central retina with the fovea was embedded in 2.25% carboxymethylcellulose (CMC) before sectioning a 93 year old retina donor tissue at 12-14 µm thickness using a Leica CM3050S (Leica, IL, USA) at -20 °C and mounting onto indium tin oxide (ITO) coated microscope slides (Delta Technologies ETC). Samples were vacuum sealed with oxygen absorbing packets and transported to Vanderbilt University on dry ice and stored in a -80 °C freezer. Before analysis, slides were brought to room temperature and dried in a vacuum desiccator for a minimum of 30 minutes. Cornea tissues were prepared from a whole eye globe from a 59 year old donor, the globe was fresh frozen and embedded in 15% fish Gelatin (Sigma Aldrich, St. Louis, MO, USA). The whole globe was then divided into two halves using a rotating cutting (Dremel 402 mandrel, Dremel, 402, Walnut Ridge, AR, USA) saw to reduce the size of the sample in order to improve section quality and reproducibility. Sections were taken around the nasal side of the mid point of the lens (were the nucleus of the lens and pupil were visible). Detailed method on this preparation can be found at (`https://www.protocols.io/view/uab-vu-biomic-preparation-of-left-fresh-frozen-eye-3byl4jd38lo5/v1`).

MALDI Q-ToF IMS Datasets

The following is based on a description by the same co-authors in Anderson et al.[1]. The MALDI matrices, 1,5-diaminonaphthalene (DAN, 15 mg) for negative ion mode and 2,5-dihydroxyacetophenone (DHA, 20 mg) (Tokyo Chemical Industry CO, Tokyo, Japan.) for positive ion mode, were applied to tissue sections using a custom designed sublimation device. MALDI IMS data were acquired with a 10 μm pixel size for the retina with a 10 μm pitch, while data for the cornea were acquired at 20 μm pixel size with a 20 μm pitch in full scan mode using a timsTOF Pro for the retina and a timsTOF Flex for the cornea, MALDI imaging platform in QTOF mode (Bruker Daltonik, Bremen, Germany), laser parameters such as laser power and beam scan for the 20 μm pitch were optimized for each experiment varying pixel size. Data were acquired with 250 laser shots per pixel and within a mass range of m/z 300-2000 for the retina and 500-2000 for the cornea. The mass spectrometer was calibrated with red phosphorus prior to data acquisition.[3]

Table 1: Table containing the specifics of our two datasets, including information on the wet-lab specifics and data footprint specifics.

**Specifics**	**Cornea**	**Retina**
Section thickness	12 μm	12-14 μm
Slide type	Poly-lysine coated ITO	Poly-lysine coated ITO
MALDI matrix	1,5-diaminonaphalene (DAN)	2,5-dihydroxyacetophenone (DHA)
Ionization mode	Negative	Positive
Pixel size	20 μm	10 μm
Instrument	Bruker MALDI timsTOF Flex	Bruker MALDI timsTOF Flex
Preprocessing	m/z aligned, calibrated and normalized by 5-95% TIC	m/z aligned, calibrated and normalized by 5-95% TIC
Mass-to-charge range	m/z 500-2000	m/z 300-2000
Data table	235,218 pixels×2,381 peaks	137,923 pixels×3,212 peaks
Variable type	32 bits int	32 bits int
Data size	2.24 GB	1.77 GB
Raw data size	6.28 GB	30.7 GB
Peak picked	Yes	Yes

Data Preprocessing

The following is based on a description by the same co-authors in Zhang et al.[4]. Data were exported from the Bruker timsTOF file format (.d) to a custom binary format for easy access and improved performance. Each pixel/frame contains between 10^4 and 10^5 centroid peaks covering the entire acquisition range, which can be reconstructed into a pseudo-profile mass spectrum using Bruker's SDK (v2.21). The dataset was m/z-aligned using six internally identified peaks (appearing in at least 50% of the pixels) through the msalign library (v0.2.0).[5,6] This step corrects spectral misalignment (drift along the m/z axis), increasing overlap between spectral features (peaks) across the experiment. Subsequently, the mass axis of the data set was calibrated using the theoretical masses of the six peaks, achieving a precision of approximately ±1 ppm. Normalization correction factors were computed following the preprocessing steps and an outlier-insensitive total ion current variant that only includes data lying between the 5th and 95th percentiles of each mass spectrum (5/95% TIC) was used for mass spectral and ion image normalization. Subsequently, an average mass spectrum based on all pixels was calculated for each dataset. Each mass spectrum was peak-picked independently of each other, producing a feature list of 2,381 peaks for the cornea and 3,212 for the retina. It is important to note that isotopic peaks were not removed prior to proceeding with the analysis. The feature lists were used to extract data into a two-dimensional matrix of shape (N x M) where N is the number of pixels and M is the number of features. An image was created by summing the intensity of a specified peak with a ± 3-5ppm extraction window. Before further analysis, the image intensity matrix is normalized using the 5/95% TIC normalization factors.

Micrographs

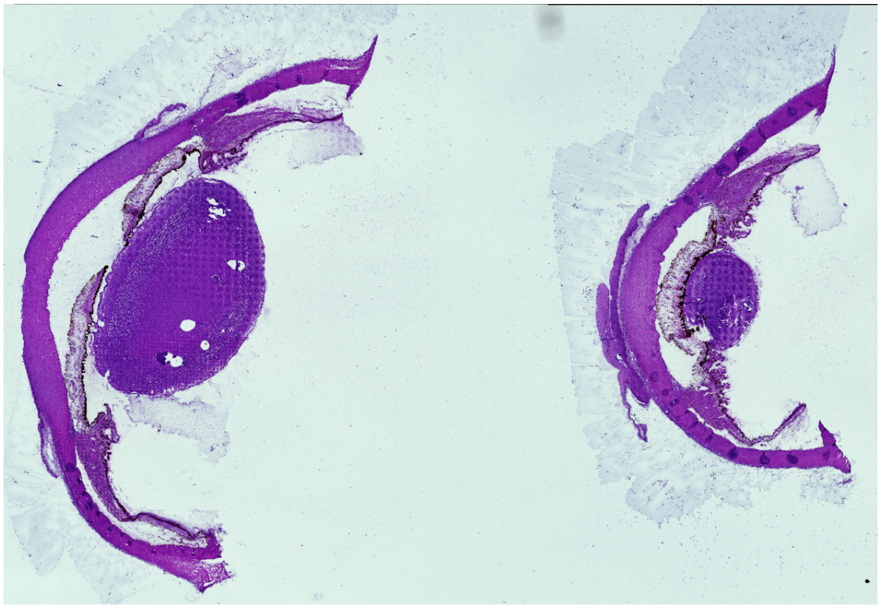

Figure S1: Human cornea H&E-stained image where fine sparse layers, *e.g.*, lens, ciliary processes, and iris, can be observed.

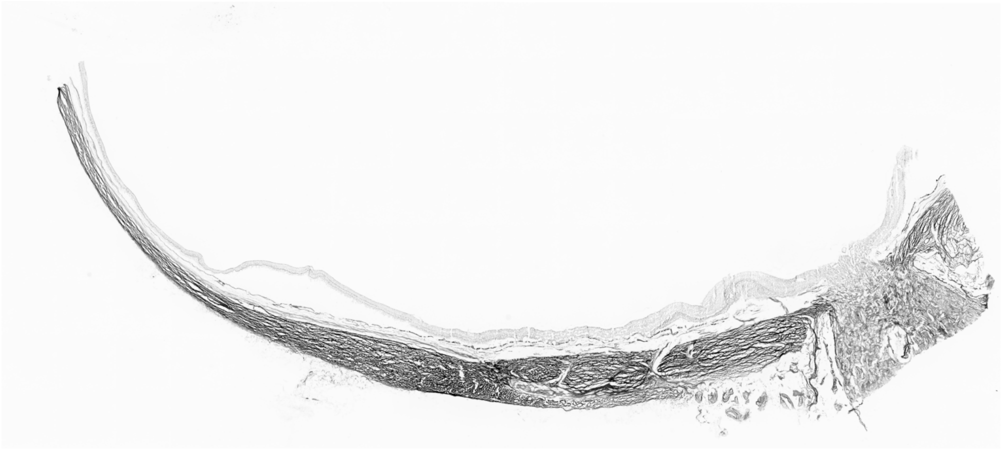

Figure S2: Human retina brightfield image revealing major and fine tissue structures including inner retina, choroid, and sclera.

Supplementary Information to the Methods

This section outlines the theoretical conditions and assumptions underlying the PCP and SPCP methods, including sparsity, incoherence, and noise-bound constraints.

Method Conditions

For PCP and SPCP, several conditions for exact or bounded retrieval are imposed, including incoherence conditions on the low-rank term, *i.e.*, $\mu(A) \leq \eta$ for some η;[7] a sparsity constraint for the sparse residuals term, *i.e.*, $\|B\|_0 \leq \gamma$ for some γ, (although this constraint can be relaxed to allow for dense noise[8]); and a size constraint (entry-wise) of the dense residuals term, *i.e.*, $\|C\|_F \leq \delta$ for some δ. Note that conditions like incoherence are difficult to verify. In this paper, we therefore assume that those conditions are satisfied. For a more elaborate sensitivity analysis with respect to these conditions, we refer the reader to prior work.[9]

Supplementary Information to the Case Studies

In this section, the rationale, parameter selection, and methodology for conducting both dimensionality reduction and noise reduction case studies are elaborated on in-depth.

Case Study 1: Focus on Dimensionality Reduction

The focus of Case Study 1 is on comparing the methods' dimensionality reduction performance. We will be using the human cornea dataset to illustrate this aspect.

To make a fair comparison of the quality of low-rank data approximations by PCA, PCP, and SPCP, would require that the low-rank data approximations are all given the same number of components, *i.e.*, the same rank, to work with. Moreso, we would like to assess these methods across a large range of possible ranks, so the challenge becomes how to obtain PCA, PCP, and SPCP low-rank approximations that are matched in rank and this for different ranks. Since PCA has the rank r as an explicit parameter, it is not hard to obtain PCA results for a specific rank at which we want to compare. More precisely,

the rank of a PCA result can be easily and uniquely set by truncating the underlying SVD. However, the rank of the B-matrix is not an explicit parameter of PCP and SPCP, and thus it is much less straightforward to retrieve PCP and SPCP results that employ a specific rank. In other words, it is complicated to construct bijective maps for PCP and SPCP, such that for PCP a unique λ-parameter value maps to a specific rank, or for SPCP a unique (θ, σ)-parameter value set yields a specific rank result. Instead, we approach the problem the other way around and perform an extensive parameter sweep for all methods to obtain the different rank results needed to drive a fair comparison.

Therefore, regarding SPCP, we execute a GPU-accelerated implementation of the SPCP algorithm on a randomly selected 10% subset (heuristically determined) of the total number of pixels in the cornea dataset, and repeat this for the 10,000 possible parameter settings of σ and θ-multipliers. The 10,000 recovered low-rank terms B_{SPCP} are saved and their rank and relative rank are depicted in Suppl. Figure S3 for each combination of the σ and θ-multipliers. The white entries in this figure correspond to trivial solutions, *e.g.*, $B_{\text{SPCP}} = 0$, which yield zero-rank approximations of the data that are not useful for dimensionality reduction purposes (not for compression or feature extraction, nor for human interpretation). In the non-white (non-trivial) parameter combinations in Suppl. Figure S3, we can observe that for an increasing θ-multiplier the rank tends to increase. This is to be expected since a larger θ-multiplier translates into a larger θ-value in SPCP's model in Equation 4, a growing need to keep the nuclear norm in the optimization's objective function low, and thus a higher barrier for variation to be captured by the sparse residuals term. In cases where the σ-multiplier and its corresponding dense residuals term D_{SPCP} are kept the same, the variation that can no longer be captured by the sparse residuals term C_{SPCP} will increasingly need to be captured by the low-rank term B_{SPCP}, resulting in an increase in the rank of that matrix. This increase in rank from close to zero up to $\sim$2400 with an increasing θ-multiplier is visible along the vertical axis of Suppl. Figure S3. We also observe that as the σ-multiplier increases, the rank goes down. This is also expected since an increased σ-multiplier value translates into a higher

value for parameter δ in Equation 4, a higher allowed amplitude for the dense residuals in D_{SPCP}, and thus less need for the low-rank term B_{SPCP} and the sparse residuals term C_{SPCP} to capture the actual variation found in the measurements of A. Regardless of whether the barrier to enter the sparse residuals term C_{SPCP} is kept the same or not by the θ-multiplier, the lack of pressure to capture what is in the measurement matrix A that comes with an increased σ-multiplier tends to result in a decreased rank of the low-rank approximation, as is visible along the horizontal axis of Suppl. Figure S3. Furthermore, it can be observed that the relationship between the σ and θ-multipliers is not linear, and that the parameters they control tend to drive SPCP's model in Equation 4 to utilize the B_{SPCP}, C_{SPCP}, and D_{SPCP} terms as communicating barrels to capture A's content. Overall, an increasing θ-multiplier and decreasing σ-multiplier correspond to higher-rank approximations, while a decreasing θ-multiplier and increasing σ-multiplier yield lower-rank approximations. Which parameter combination and corresponding decomposition is best for a practical application depends on the particular needs of that application: the lower the rank, the smaller the dimensionality of the approximation of the measurement set, the higher the compression ratio, but the less degrees-of-freedom the model has; the higher the rank, the more signal patterns are captured, but the higher the dimensionality of the approximation of the measurements and the worse the compression ratio. Finally, it is useful to point out the smoothness of the rank-space depicted in Suppl. Figure S3, which essentially depicts the content of the low-rank term B_{SPCP}. The smooth transitions suggest that the rank-space is differentiable and thus could be directly exploited for hyper-parameter optimization towards particular applications and constraints in future research.

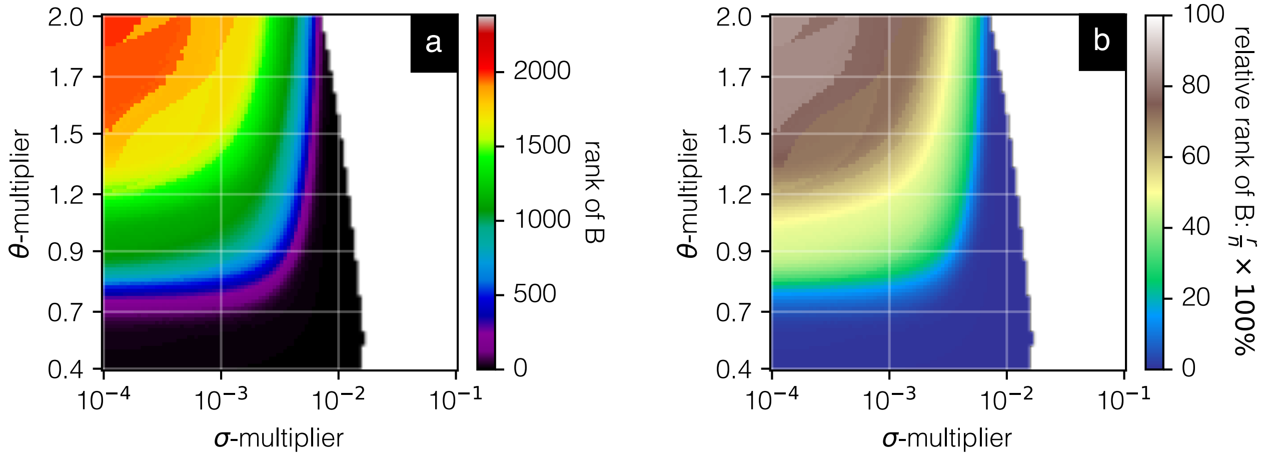

Figure S3: Recovered rank (a) and relative recovered rank (b) of the low-rank term (B) for the cornea dataset in function of the SPCP algorithm's θ- and σ-multiplier parameters. The relative recovered rank is taken with respect to the maximal possible matrix rank, *i.e.*, the $\min(m, n)$, where $B \in \mathbb{R}^{m \times n}$. White entries in the heat maps correspond to trivial solutions, *i.e.*, rank $= 0$. The rank and relative rank increase smoothly when moving from low θ and high σ values to high θ and low σ values. With the compression application in mind, we are interested in (θ, σ)-pairs that yield a relative rank below 20%.

Secondarily, for PCP results, a GPU-accelerated implementation of the PCP algorithm is run on the same 10% subset of pixels, and repeated for $2,000$ distinct values of the λ-multiplier. The 2000 recovered low-rank terms B_{PCP} are also saved for further processing. Third, PCA is run for all possible rank values and its corresponding recovered low-rank terms B_{PCA} are also stored to disk.

In order to match up PCA, PCP, and SPCP results in terms of rank, the rank of the SPCP results is chosen as a reference point. Next, for each SPCP parameter combination, the closest approximation of the same rank was sought within the PCP and PCA results. For selecting a corresponding PCP result, an additional constraint was set in that its rank must be equal or higher than the SPCP rank. Also, a second constraint was imposed, necessary to achieve a one-to-one map, stating that out of all PCP results with the rank we are looking for, the PCP solution with the highest λ value is selected. The latter constraint is necessary as different λ settings might yield the same rank in their solutions, and this rule ensures that the solution with largest nuclear norm is chosen. In conclusion, for each SPCP parameter value set (and thus B_{SPCP} approximation of A), we obtain a corresponding and unique PCP

approximation B_{PCP} and PCA approximation B_{PCA} of that same A, all with the same rank enabling comparison of their content.

Case Study 2: Focus on Noise Reduction

Contrary to Case Study 1, here we do not consider all possible combinations of the θ and σ-multipliers, but instead restrict ourselves to five distinct parameter settings (Case 1 through 5) that are representative of different solution areas within the parameter space (see Suppl. Figure S4). Similar to the first case study, the low-rank approximations obtained at these five locations within the parameter space for SPCP (B_{SPCP}) are matched to low-rank approximations provided by PCP (B_{PCB}) and PCA (B_{PCA}), possessing the same rank.

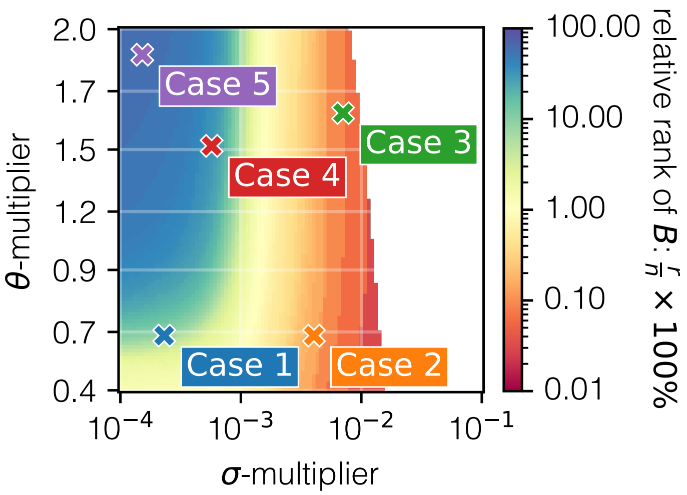

Figure S4: Varying parameter settings lead to different results for each method for the retina dataset. In Case Study 2, quantitative and qualitative results are explore for 5 cases, each representing a different part of the solution space. The different cases are: (Case 1) low rank (r=126) through low θ-multiplier and low σ-multiplier; (Case 2) very low rank (r=5) through low θ-multiplier and high σ-multiplier; (Case 3) very low rank (r=3) through high θ-multiplier and high σ-multiplier; (Case 4) middle rank (r=1018) through middle θ-multiplier and middle σ-multiplier; (Case 5) high rank (r=1825) through high θ-multiplier and low σ-multiplier.

For each of these five parameter cases, we also investigate effects local to a specific m/z bin, examining ion distributions that exhibit interesting relevant aspects such as being dominated by low or high ion intensities or the presence of sparse spatial structures. This selection

of six m/z-bins consists of (1) m/z 601.53, a representative of ion species whose distributions exhibit sparse spatial structures; (2) m/z 1007.01, an example of ion distributions with only low intensity values; (3) m/z 790.52, a representative of ion images with low to average intensity values; (4) m/z 666.43, representing ion species with average intensity values; (5) m/z 554.57, an example of ion images with relatively high intensity values without strong outliers (here considered to be a spatially sparse features); and (6) m/z 591.01, representing high intensity ion distributions with strong outliers.

Supplementary Information to the Comparison Metrics

Below, we first formulate a content-based set of metrics, capturing the overlap between PCA, PCP, and SPCP captured subspaces. Then, we formulate a set of non-negativity based metrics as heuristics for how strongly the different low-rank approximations differ from the physical reality of ion counts.

Content-based Metrics: Metrics Reporting overlap in Recovered Column and Row Subspaces

Let us consider two low-rank approximations of the same measurement set A, namely a low-rank matrix $B_{\text{Method 1}}$ obtained by applying a Method 1 and another matrix $B_{\text{Method 2}}$ provided by a Method 2. Even if Methods 1 and 2 were to capture largely the same subspace with their low-rank approximations of the data, there is little reason for the basis vectors captured in $B_{\text{Method 1}}$ and $B_{\text{Method 2}}$ to be identical, and so it is not really an option to compare components directly between methods. Therefore, we need a method to capture the overlap between two recovered subspaces, while remaining insensitive or invariant to the specific basis vector entries. Below, we formulate a principal angles/subspace similarity approach that uses the singular value decomposition to find correspondence between two sets of basis vectors.[10]

Furthermore, a r-rank matrix B of size $m \times n$ can be written as its singular value de-

composition, *i.e.*, the product of a matrix U of size $m \times r$, a diagonal matrix S of size $r \times r$ and the transpose of a matrix V of size $n \times r$, such that $B = USV^T$. As such, we can examine the subspace overlap along the spatial domain, *i.e.*, U, separately from the subspace overlap along the spectral domain, *i.e.*, V. The spatial subspaces are provided by $U_{\text{Method 1}}$ and $U_{\text{Method 2}}$, and report the "component images" encoded into the columns of $B_{\text{Method 1}}$ and $B_{\text{Method 2}}$ respectively. These images describe where in the tissue a certain component is active. The spectral subspaces are provided by $V_{\text{Method 1}}$ and $V_{\text{Method 2}}$, and report the "component spectra" encoded into the rows of $B_{\text{Method 1}}$ and $B_{\text{Method 2}}$ respectively. These pseudo-spectra describe which m/z bins or features in the IMS data are involved in a certain component.

The method we will use to assess overlap between two sets of basis vectors is to calculate a singular value decomposition of the product between the transpose of one set of basis vectors and the other set of basis vectors. The result reflects the alignment of the provided bases, and we calculate two metrics using this approach. Metric α reports the overlap between the (spatial) column subspaces of $B_{\text{Method 1}}$ and $B_{\text{Method 2}}$, while metric β reports the overlap between the (spectral) row subspaces of $B_{\text{Method 1}}$ and $B_{\text{Method 2}}$. These metrics are calculated as follows:

$$\begin{aligned} &U_c \Sigma_c V_c^T = \text{svd}(U_{\text{Method 1}}^T U_{\text{Method 2}}), \\ &\alpha = \frac{1}{r}\mathbf{tr}(\Sigma_c), \end{aligned} \tag{1}$$

and

$$\begin{aligned} &U_r \Sigma_r V_r^T = \text{svd}(V_{\text{method 1}}^T V_{\text{method 2}}), \\ &\beta = \frac{1}{r}\mathbf{tr}(\Sigma_r). \end{aligned} \tag{2}$$

Singular values, here captured in Σ_c and Σ_r, close to 1 indicate bases that are captured in both subspaces, while singular values close to 0 correspond to orthogonal basis vectors that differ between the subspaces. To report the closeness of the mutually recovered column and row subspaces as a single number, we define the mean of singular values as our final metric. The latter ensures that every singular value is equally weighted.

Non-negativity Metrics

Ion intensity values measured by mass spectrometry are inherently non-negative. Therefore, any low-rank approximation of an IMS dataset that leans substantially on negative values in its underlying components might be mathematically a valid decomposition into vectors (potentially suited, *e.g.*, for compression), but it is unlikely to be a good representation of the underlying biological trends present in the data (*i.e.*, not suited for human interpretation). One could set non-negativity as a constraint for a low-rank decomposition of IMS data, as in, *e.g.*, non-negative matrix factorization, but in this work the goal is not to enforce non-negativity, but rather to focus on the sparsity related issues that come with using PCA in mass spectrometry data analysis, and PCA does not have such a non-negativity constraint built in. Moreso, with the focus on addressing sparse signal capture, none of the three models explored here optimize over a non-negative manifold or have a non-negativity constraint. Since we know molecular and ion species at a particular tissue location are either not present (zero ion count) or present (positive ion counts), and negative intensity counts have no physical meaning, we can use the presence of negative intensity values in these methods' low-rank components and approximations as a heuristic for an approximation's deviation from instrumental physics and biology. Deviations of a method's low-rank approximation from a non-negative model are captured using three different metrics: the percentage of negative entries in the low-rank term B of each method, the total sum of those entries, and the mean of those same entries. They reflect respectively the relative number of negative (and therefore non-biological) values in a low-rank approximation, the total magnitude of this mathematically correct, yet biologically improbable deviation, and the average magnitude of the deviation. Note that many more such metrics could be constructed. This particular set of metrics is selected for their simplicity and independence from the three methods explored here and their underlying models.

Supplementary Information to the Results of Case Study 1

This section explores how different parameter settings influence the occurrence, magnitude, and distribution of negative entries in the low-rank approximations, evaluating their impact on interpretability.

Negative Entries in the Low-Rank Approximation

A low-rank approximation of a measurement set that is inherently non-negative can carry negative entries and still be useful for downstream analysis that does not require a physical interpretation of its components (*e.g.*, for data compression or preceding a supervised machine learning model). However, if the goal is to obtain a low-rank approximation of these non-negative measurements that can be biochemically interpreted, physical feasibility of the recovered components becomes more important. One could enforce non-negativity on a low-rank approximation of a dataset, as in non-negative matrix factorization.[11] However, here we are interested in gauging, when methods that do not enforce non-negativity are applied to inherently non-negative data, whether an added sparse residuals term C as in PCP and SPCP impacts the non-negativity of the low-rank approximation, and thus the physical feasibility and interpretability of the components and underlying trends retrieved from the data. Since negative ion counts are not physically possible, the percentage, sum, and mean of negative entries in a method's low-rank approximation that is not constrained to be non-negative could be used as heuristics to quantify that method's tendency to deviate from a physically feasible low-rank model. The results in terms of negative entries for PCA, PCP, and SPCP approximations are provided in the Supplementary Information, and discussed in-depth in sections dedicated to the percentages, sums, and means of those entries.

The results of Supplementary Figures S5, S6, and S7 suggest that, at least for this cornea dataset, SPCP outperforms both PCP and PCA for almost all parameter settings. Concurrently, the rank of the low-rank approximation term of SPCP and PCP seems positively correlated to the number of negative entries, *i.e.*, a higher rank will lead to more negative

entries. However, the average negative entry will be small in magnitude. Our hypothesis is that this phenomenon is caused by noise being captured by the low-rank approximation term.

Percentage of Negative Entries

Supplementary Figure S5 shows the percentage of negative entries in the low-rank term B of SPCP (panel a), PCP (panel b), and PCA (panel c), and this for all explored parameter settings. The value at a particular location in panel (a) can be directly compared to the value found in panels (b) and (c) at that same location. For SPCP, we observe that for all parameter settings less than 10 % of the entries in B_{SPCP} contain negative values, while for PCP and PCA this percentage goes up to 18 %. While SPCP outperforms PCP and PCA for this data set for nearly all considered parameter values, PCP also outperforms PCA, but only for high-rank cases. Furthermore, in the region of approximations of relatively low rank (*i.e.*, below 20 %; see Suppl. Figs. S3b and S5c), we see that for high σ-multiplier values PCA and SPCP both produce a similar percentage of negative entries. This could indicate that in cases where the deviation from dataset A is allowed to be larger, that allowance for dense residuals could translate into less need to introduce negative entries into the low-rank approximation. Since PCA and SPCP both have a dense residuals term D, this relieves their low-rank term B and sparse residuals term C from having to capture non-structured, non-sparse variation in the data, and presumably could allow the low-rank approximations to stay closer to the non-negative physical reality. While we have no proof of this hypothesis, it does seem to be confirmed by the relatively worse PCP results in Suppl. Figure S5b. Since the PCP model does not have a dense residuals term D, its low-rank term B and sparse residuals term C are forced to absorb non-structured, non-sparse variation in the dataset (by PCP's constraint that $A = B + C$), which could be the reason that PCP has many more negative entries in its low-rank approximations than SPCP has. Finally, we remark that a very similar smooth pattern can be observed in Suppl. Figure S5 as in the rank

pattern of Suppl. Figure S3. This suggests that an increasing percentage of negative entries tends to correlate to increasing rank. This could mean that as more components are added to the low-rank approximation, there are more degrees of freedom to do a mathematically optimal approximation that nevertheless deviates from physical feasibility. It also suggests that if physical feasibility is important, a lower-rank approximation is probably more suitable than a higher-rank approximation, at least for cornea data. Overall, for all three methods, rank of their low-rank approximation seems to be positively correlated to the percentage of negative entries. Furthermore, in all tested parameter settings, SPCP has a smaller number of negative entries in the components of its low-rank approximations than PCP and PCA. Thus, SPCP tends to deliver more physically interpretable low-rank approximations than PCP and PCA, at least for this dataset.

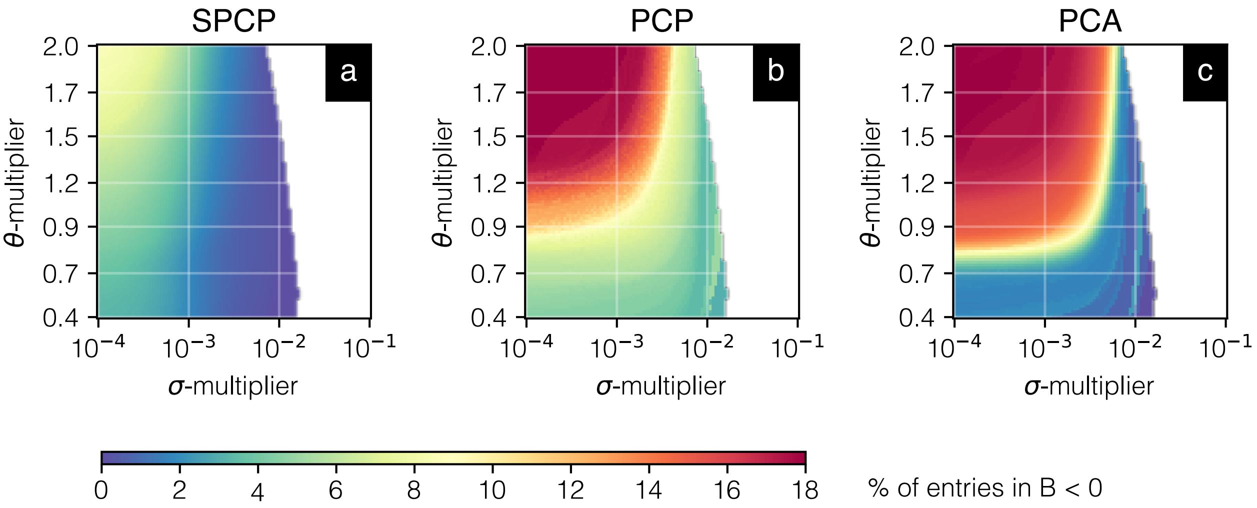

Figure S5: Percentage of negative entries in the low-rank term B of SPCP (a), PCP (b), and PCA (c). The percentage is calculated for each B_{PCA}, B_{PCP}, and B_{SPCP} that are rank matched for every θ and σ-multiplier parameter set. It reports the ratio (in %) of the number of negative entries in B (*i.e.*, $B_{ij} < 0$) to the total number of matrix entries (*i.e.*, mn for $B \in \mathbb{R}^{m \times n}$). A lower percentage of negative entries corresponds to a more physically feasible and interpretable approximation of the IMS data. For this dataset, SPCP seems to outperform both PCP and PCA for most of its parameter settings, requiring much less negative entries in its low-rank approximations to achieve the same rank and reduction of dimensionality.

Sum of Negative Entries

After investigating the number of negative entries in the different low-rank approximations, there is also value in assessing the magnitude of these deviations from physical feasibility. The sum of negative entries in B reflects the total dataset-wide magnitude of the deviation from easy interpretability of an approximation's components. We plot this sum for every examined parameter setting in Suppl. Figure S6.

For this data set, and similar to our observations regarding the percentage in Suppl. Figure S5, SPCP seems to outperform both PCP and PCA, for most of its parameter settings roughly by a factor 2 to 10. We also see that for PCP (Suppl. Figure S5b) with increasing rank the sum of negativity increases as well, while the inverse is observed for PCA (Suppl. Figure S5c). For rank-1 solutions (bottom-right), the negativity of all methods is comparable, while for low-rank settings (*i.e.*, below 20 % relative rank), SPCP's magnitude of negativity is the least of all three methods. Finally, we distinguish discrete steps in the very low-rank areas for PCA (bottom-right). This could be originating from high-intensity sparse features in the dataset being captured by the first few components of a low-rank approximation by PCA, and subsequent principal components trying to 'off-set' these components with a lot of negative entries to 'fulfill' PCA's orthogonality constraint (without the availability of a sparse residuals term). In conclusion, SPCP tends to accumulate the least total negative magnitude in its low-rank approximations when compared to PCP and PCA, and for this dataset we observe a positive correlation between the sum of negative entries and the rank of the approximations. This seems to further confirm that, given the same rank or dimensionality to work with, SPCP delivers more physically interpetable low-rank approximations than PCP and PCA, at least for this dataset.

Mean of Negative Entries

The sum of negative entries gives an idea of the total deviation of a low-rank approximation from physical interpretability for inherently non-negative data. The percentage of negative

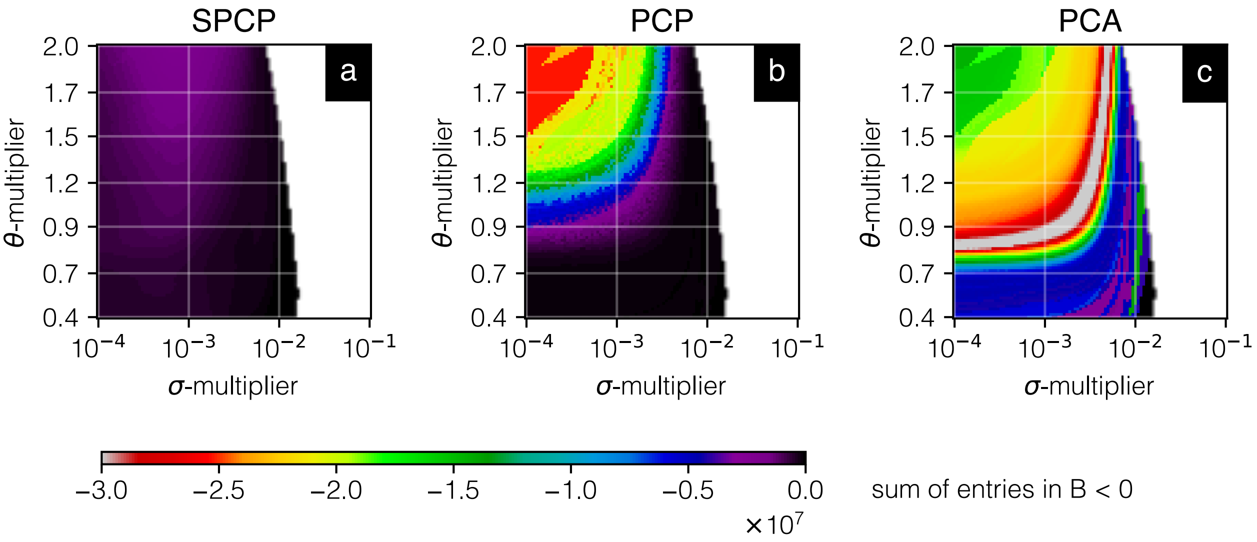

Figure S6: Sum of negative entries in the low-rank term B of SPCP (a), PCP (b), and PCA (c). The sum is calculated for each B_{PCA}, B_{PCP}, and B_{SPCP} that are rank matched for every θ and σ-multiplier parameter set. The sum reports the magnitude of the number of negative entries in B (*i.e.*, $B_{ij} < 0$). A value closer to zero corresponds to a more physically feasible and interpretable approximation of the IMS data. For this dataset, and similar to Suppl. Fig. S5, SPCP seems to outperform both PCP and PCA, for most of its parameter settings roughly by a factor 2 to 10.

entries gives an impression of how widespread this deviation is. Now, the mean of negative entries can show us whether only few large magnitude negative values are present or whether there is a large number of smaller magnitude negative values. Supplementary Figure S7 shows the mean of negative entries in the low-rank term B of SPCP (panel a), PCP (panel b), and PCA (panel c), and this for all explored parameter settings. This figure can be interpreted in a manner similar to Suppl. Figs. S5 and S6.

For SPCP, a slightly higher mean is observed for σ-multiplier values close to 10^{-2}. This might be an artifact of the relatively lower number of negative entries in that area due to the very low-rank approximations achieved there. For PCP, one can discern again a correlation with the rank, albeit it a very slight one. In general, the mean negative entries for SPCP and PCP seem to be low compared to PCA. For PCA, we observe in the low rank region, *i.e.*, where the relative rank is below 20%, a relatively high mean of the negative entries, indicating that PCA's low-rank approximation is deviating quite a lot from a physically

interpretable decomposition. Overall, the average negative entries seem the smallest for SPCP and PCP in comparison to PCA, at least for this dataset.

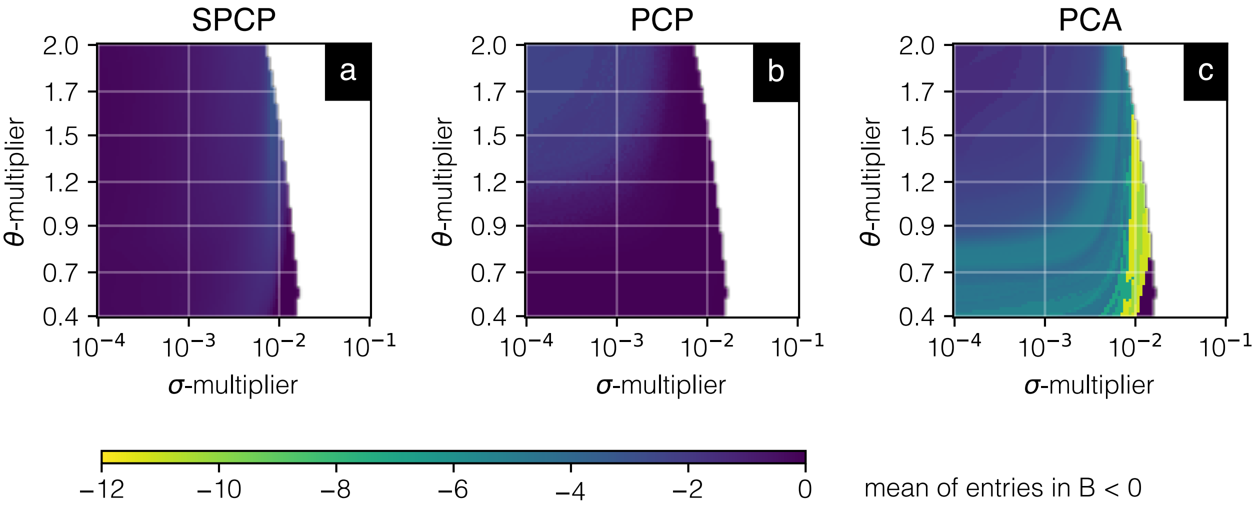

Figure S7: Mean of negative entries in the low-rank term B of SPCP (a), PCP (b), and PCA (c). The mean is calculated for each B_{PCA}, B_{PCP}, and B_{SPCP} that are rank matched for every θ and σ-multiplier parameter set. The mean reports the average magnitude of negative entries in B (*i.e.*, $B_{ij} < 0$). A value closer to zero corresponds to a more physically feasible and interpretable approximation of the IMS data. For this dataset, and similar to Suppl. Figs. S5 and S6, SPCP seems to outperform both PCP and PCA, for most of its parameter settings except for regions of 'large' σ-multiplier values. In these latter regions, SPCP outperforms PCP in terms of percentage and sum of negative entries in its low-rank approximation and thus has fewer negative values to begin with, but they do tend to be of larger average magnitude compared to PCP.

Supplementary Information to the Results of Case Study 2

This section investigates how ion intensity is distributed among the decomposition terms (B, C, and D) for specific parameter settings and m/z-bins, and how these affect denoising and biological signal preservation.

We explore the residuals terms delivered by PCA, PCP, and SPCP for five select parameter settings:

- (Case 1) low rank B (r=126) through low θ-multiplier and low σ-multiplier;
- (Case 2) very low rank B (r=5) through low θ-multiplier and high σ-multiplier;

- (Case 3) very low rank B (r=3) through high θ-multiplier and high σ-multiplier;
- (Case 4) middle rank B (r=1018) through middle θ-multiplier and middle σ-multiplier;
- (Case 5) high rank B (r=1825) through high θ-multiplier and low σ-multiplier.

First, we compare the different matrix terms in general, using their element-wise histograms across all m/z bins to better understand how parameter settings influence the distribution of measured ion intensity among the different terms. Then, we compare the element-wise histograms within the scope of specific m/z bins to examine the impact of parameter settings on the ion species-specific intensity distributions. In the context of noise removal/reduction, we investigate how different parameter settings can impact the spatial distributions of the residuals terms' images. To this end, we provide a visualization of a measured ion image and its decomposition into low-rank, sparse, and dense images for the same set of m/z bins and parameter settings as before, albeit cropped to a sub-area within the retina for easier viewing (see Suppl. Figure S8). Finally, we demonstrate for two ion species, how the PCA, PCP, and SPCP methods decompose their measured ion images into a low-rank component, generally carrying tissue signal, a sparse component, sometimes carrying signal (*e.g.*, from small tissue structures), and a dense component that generally carries noise and can be removed. The comparison can be helpful in providing intuition to the reader to decide for their own datasets whether a sparse-signal-aware alternative to PCA, such as PCP or SPCP, can be useful for dimensionality and/or noise reduction purposes.

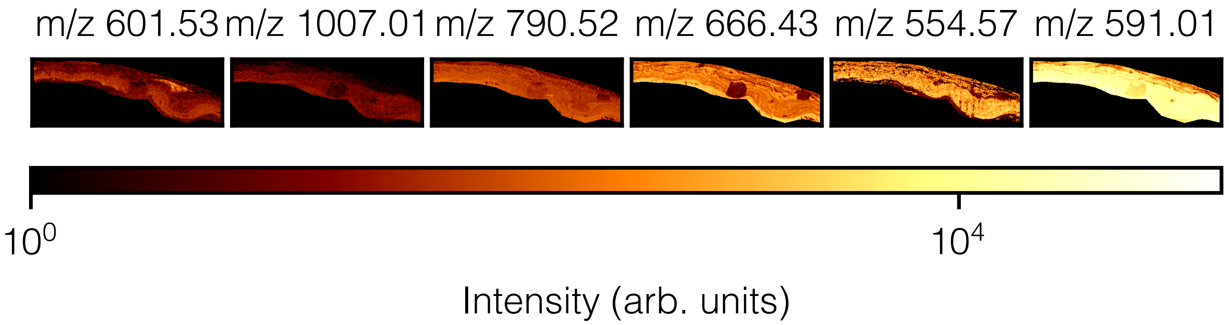

Figure S8: Six distinct m/z bins: m/z 601.53 (ion image with sparse spatial structures); m/z 1007.01 (low intensity ion image); m/z 790.52 (low to average intensity ion image); m/z 666.43 (average intensity ion image); m/z 554.57 (high intensity ion image without strong outliers); and m/z 591.01 (high intensity ion image with strong outliers).

Ion Intensity Distribution Among Terms

For Case 1 (low rank B), one can observe that the B and C terms of PCP and SPCP seem to match closely. This is expected, as when the σ-multiplier is low, very little to no energy can be captured by SPCP's D term. This makes SPCP's model (Equation 4) approximate PCP's model (Equation 3), leading to the match observed in the first row of Suppl. Figure S9. In noise reduction use cases, the measurement variation that is not structured and therefore cannot be captured well by a low-rank approximation B can be labeled as noise, suggesting that the dense residuals in D can be thrown out. Setting the σ-multiplier too high, as in Case 3 (very low rank B), releases SPCP from the need for $B + C$ to closely approximate A and results in a high amount of measurement energy to be captured by the dense residuals term D. As σ and its corresponding δ grow larger, there is no inherent mechanism preventing higher intensity biological signals from ending up in D. If biological signal variation is captured by D, removing D for noise reduction purposes is clearly undesirable. Overall, the presence of the D term gives SPCP decidedly an advantage over PCP, allowing tighter low-rank modeling (see Case Study 1 and Suppl. Figs. S5-S7). However, Case 3 illustrates that, in noise reduction use cases, if σ is set too high, there is a risk that genuine biological signal is removed. Case 1 and 3 together indicate that, when using SPCP, it is important to keep σ (and the corresponding δ) large enough to obtain the tighter modeling advantages that come

with the presence of a dense residuals term, but small enough to avoid that higher intensity biological signals start entering D and are removed as noise. For SPCP in Case 2 (very low rank B), two peaks (in blue) can be observed at roughly -2000 and +2000 ion intensity in the histogram of the D term (more clearly visible in the D zoom-in). These are the result of the SPCP model (Equation 4) putting an upper intensity bound δ on the Frobenius norm of the difference between A and $B + C$, effectively clipping intensity variations that are not captured by the low-rank and sparse residuals terms to a maximum intensity. A similar set of clipping peaks can be observed in Case 4, albeit at ion intensities closer to zero since Case 4 uses a lower σ (and thus lower δ) than Case 2. Case 2 and 4 indicate that the σ-multiplier or direct specification of the δ-parameter in Equation 4 can be used to define an ion intensity threshold, below which dense measurement variation can be captured by D and be removed as noise, and above which dense measurement variation is forced to be captured by either the low-rank approximation term B or the sparse residuals term C, both of which tend to be kept as non-noise variation.

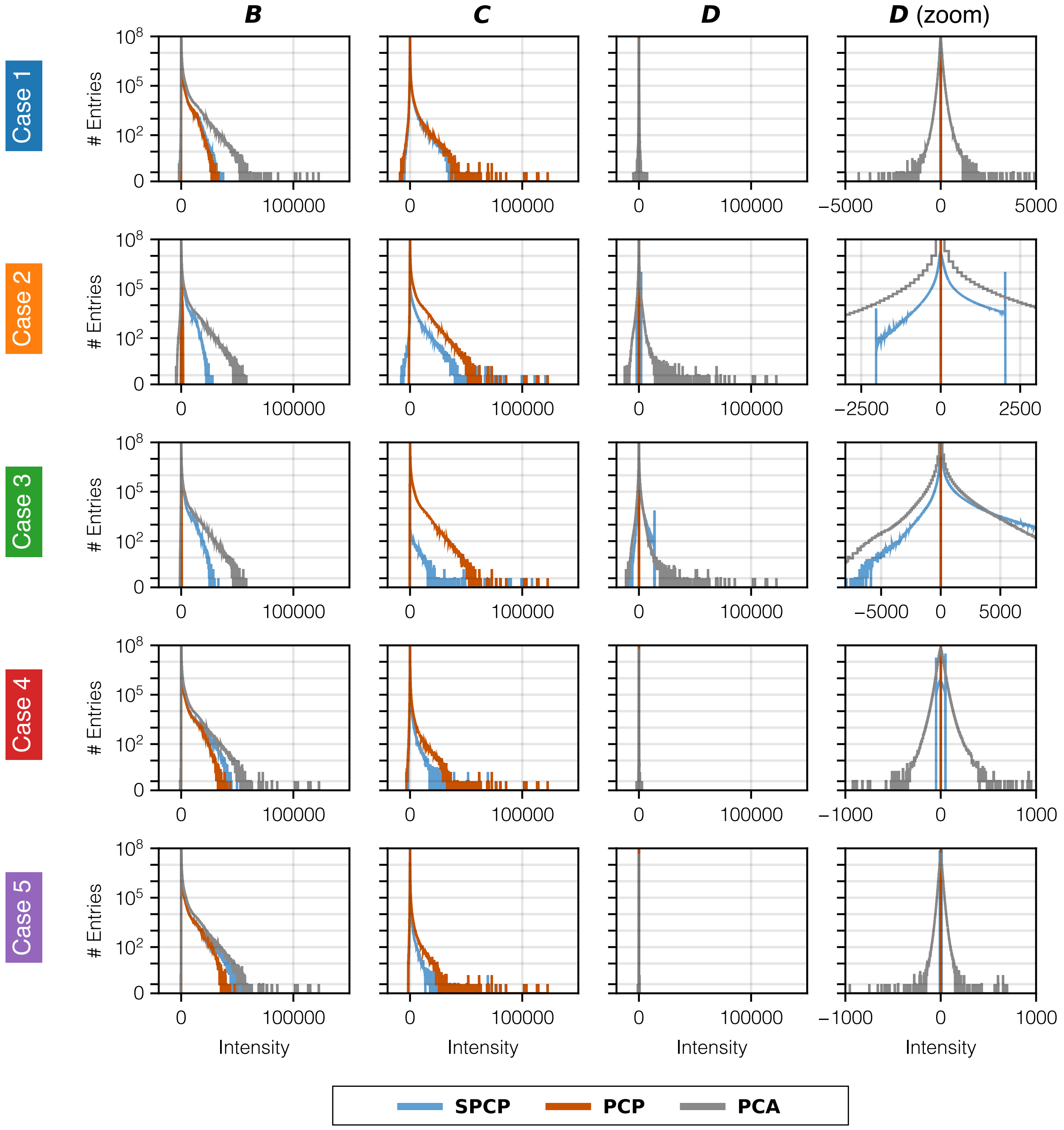

Figure S9: Global entry-wise intensity histograms for the B, C, and D terms delivered by SPCP, PCP, and PCA. For D an extra zoomed-in plot is shown. The impact of the different parameter settings is primarily visible in the C and D columns. We observe, *e.g.*, that the σ-multiplier for SPCP controls a dense residuals term as an "escape valve" for non-low-rank non-sparse variation in the measurements. It has a large influence on how the energy is distributed between C and D, and as such can be an extra handle to make C retrieve truly sparse patterns from the data.

Ion Species-Specific Effects

In the panels of Supplementary Figure S10, we show four histogram traces. We see in gray what remains after PCA's low-rank approximation, namely D_{PCA} (there is no C_{PCA}). In red, we see what remains after PCP has extracted its low-rank approximation, namely C_{PCP} (there is no D_{PCP}). In principle, C_{PCP} is optimized to capture sparse patterns. However, the red C_{PCP} traces consistently being wider than the blue C_{SPCP} profiles for the same data suggest that the lack of a dense residuals term D_{PCP} in PCP is a real impediment and forces C_{PCP} to capture more than just sparse variation. For SPCP, two traces are shown because what remains after SPCP's low-rank approximation is captured by $C_{\text{SPCP}} + D_{\text{SPCP}}$, *i.e.*, the sum of the blue and the yellow trace. The yellow trace represents non-low-rank non-sparse (dense) residuals and its content is usually suited for removal and denoising. Whether to remove the blue variation as noise depends on whether sparse features are important for the downstream analysis.

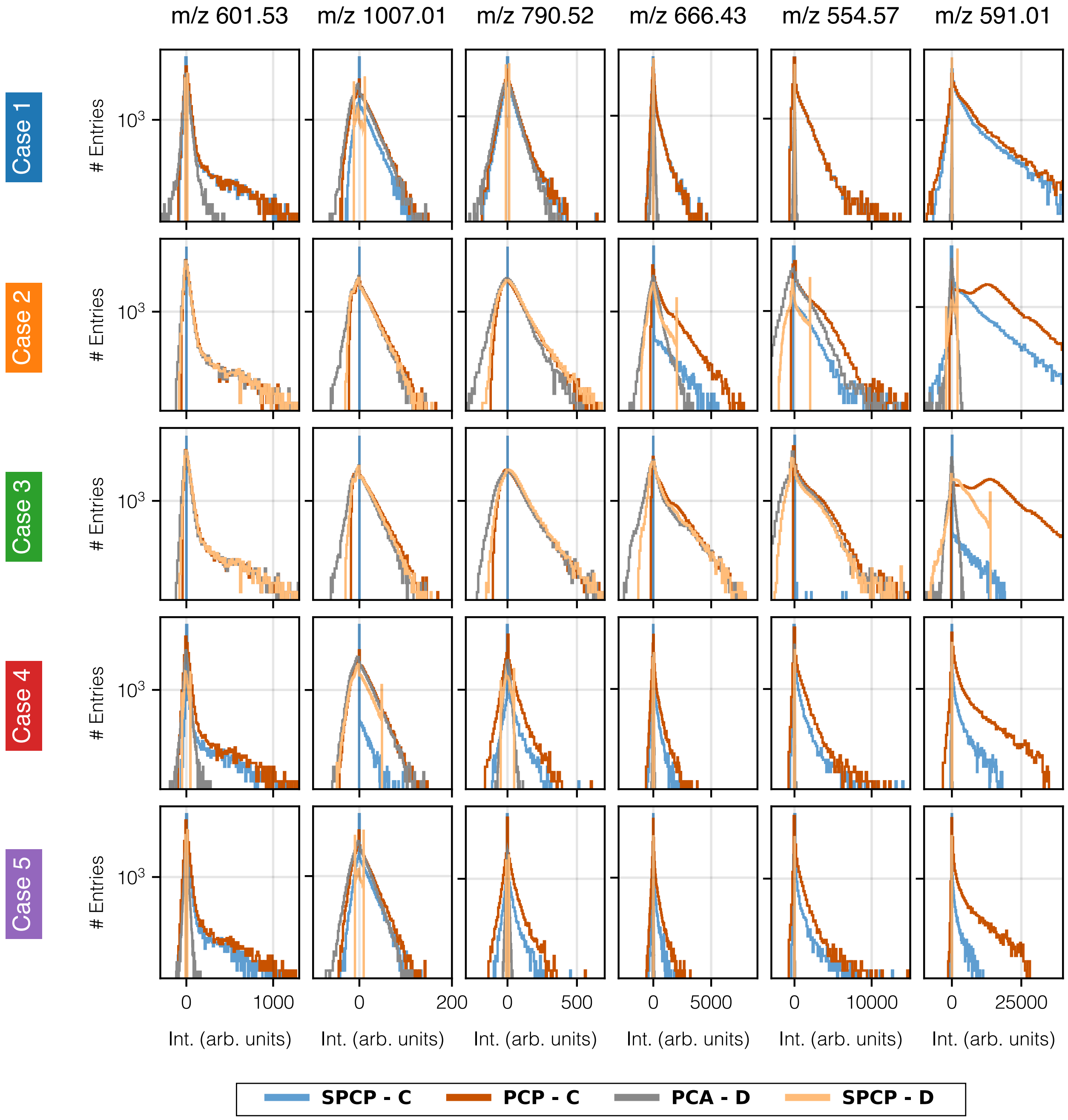

Figure S10: Mass-to-charge-specific entry-wise (for the whole data matrix) intensity histograms for the C and D terms delivered by SPCP, PCP, and PCA. The distribution of energy among C and D is examined for six distinct m/z bins: m/z 601.53 (ion image with sparse spatial structures); m/z 1007.01 (low intensity ion image); m/z 790.52 (low to average intensity ion image); m/z 666.43 (average intensity ion image); m/z 554.57 (high intensity ion image without strong outliers); and m/z 591.01 (high intensity ion image with strong outliers). Different parameter settings lead to different residuals distributions local to specific ion species, and some of these effects are tied to the nature of the ion species' intensity level (low versus high intensity ions) and spatial distribution (*e.g.*, sparse features or not). Overall, residuals of PCP and SPCP tend to contain less negative intensity values, suggesting less overestimation of the mass spectral signals in these methods' low-rank approximations.

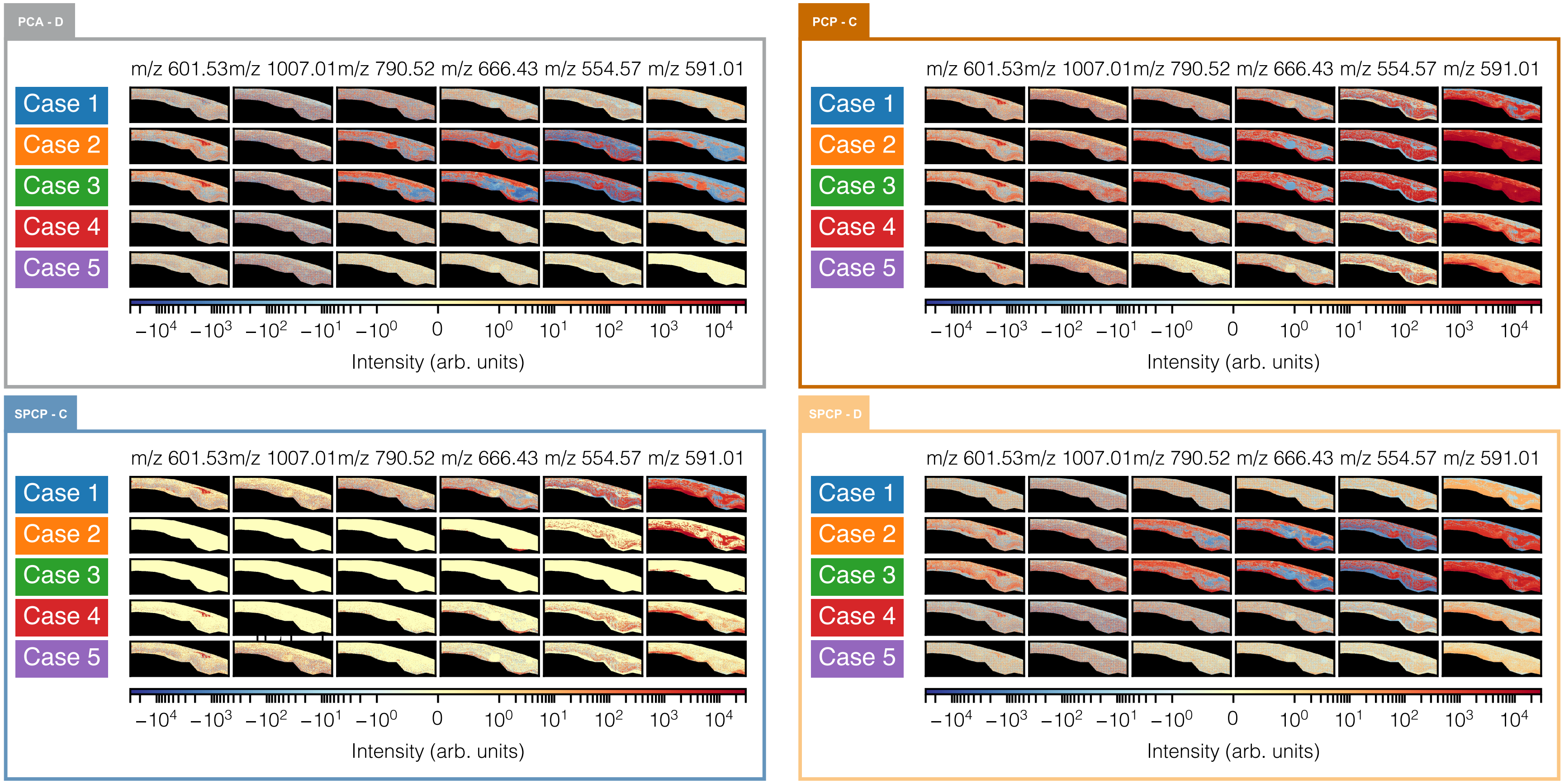

Figure S11: Images of the content of the sparse and dense residuals, *i.e.*, C and D terms, of the retina dataset for different m/z bins under different conditions for PCA, PCP and SPCP. Different parameter settings tend to lead to different results. One notable observation is that despite PCP's model trying to make the content of its C term contain sparse variation, the lack of a D term in PCP means that the C term nevertheless needs to absorb a lot of non-sparse variation as well (reflected by a lot of non-zero values in the 'PCP-C' panel, top-right). Since SPCP's model has an explicit D term for any variation that does not fit its B or C terms, SPCP's C term can be dedicated to capturing sparse variation (reflected by a lot of values close to zero in the 'SPCP-C' panel, bottom-left). The discrepancy between PCP's C and SPCP's C seems to suggest that SPCP is better at capturing genuine sparse variation.

References

(1) Anderson, D. M.; Kotnala, A.; Migas, L. G.; Patterson, N. H.; Tideman, L. E.; Cao, D.; Adhikari, B.; Messinger, J. D.; Ach, T.; Tortorella, S., et al. Lysolipids are prominent in subretinal drusenoid deposits, a high-risk phenotype in age-related macular degeneration. *Frontiers in ophthalmology* **2023**, *3*, 1258734.

(2) Anderson, D. M.; Messinger, J. D.; Patterson, N. H.; Rivera, E. S.; Kotnala, A.; Spraggins, J. M.; Caprioli, R. M.; Curcio, C. A.; Schey, K. L. Lipid landscape of the human retina and supporting tissues revealed by high-resolution imaging mass spectrometry. *J. Am. Soc. Mass Spectrom.* **2020**, *31*, 2426–2436.

(3) Sládková, K.; Houška, J.; Havel, J. Laser desorption ionization of red phosphorus clusters and their use for mass calibration in time-of-flight mass spectrometry. *Rapid Commun. Mass Spectrom.* **2009**, *23*, 3114–3118.

(4) Zhang, Y.; Huang, L.; Pillar, N.; Li, Y.; Migas, L. G.; Van de Plas, R.; Spraggins, J. M.; Ozcan, A. Virtual Staining of Label-Free Tissue in Imaging Mass Spectrometry. *arXiv e-prints* **2024**, arXiv–2411.

(5) Migas, L. G. msalign: Spectral alignment based on MATLAB's 'msalign' function. `https://github.com/lukasz-migas/msalign`, 2024.

(6) Monchamp, P.; Andrade-Cetto, L.; Zhang, J. Y.; Henson, R. Signal processing methods for mass spectrometry. *Systems Bioinformatics: An Engineering Case-Based Approach, Artech House Publishers* **2007**,

(7) Candès, E. J.; Recht, B. Exact matrix completion via convex optimization. *Found. Comput. Math.* **2009**, *9*, 717–772.

(8) Ganesh, A.; Wright, J.; Li, X.; Candes, E. J.; Ma, Y. Dense error correction for low-rank

matrices via principal component pursuit. IEEE international symposium on information theory. 2010; pp 1513–1517.

(9) Moens, R. M.Sc. Thesis. On the Atoms of Robustness: Robust Matrix Decomposition for Spectral Imaging. Delft University of Technology, 2021.

(10) Björck, A.; Golub, G. H. Numerical methods for computing angles between linear subspaces. *Math. Comput.* **1973**, *27*, 579–594.

(11) Lee, D. D.; Seung, H. S. Learning the parts of objects by non-negative matrix factorization. *Nature* **1999**, *401*, 788–791.
